# Exploring metabolic engineering design principles for the photosynthetic production of lactic acid by *Synechocystis* sp. PCC6803

**DOI:** 10.1186/1754-6834-7-99

**Published:** 2014-06-26

**Authors:** S Andreas Angermayr, Aniek D van der Woude, Danilo Correddu, Angie Vreugdenhil, Valeria Verrone, Klaas J Hellingwerf

**Affiliations:** 1 Molecular Microbial Physiology Group, Swammerdam Institute for Life Sciences, University of Amsterdam and Netherlands Institute of Systems Biology, Science Park 904, 1098 XH Amsterdam, The Netherlands; 2 Photanol BV, Science Park 408, Amsterdam, The Netherlands

**Keywords:** Cyanobacteria, L-lactic acid production, Bioplastic, Metabolic engineering, Microbial cell factory, Lactate dehydrogenase, Pyruvate kinase, Control coefficient

## Abstract

**Background:**

Molecular engineering of the intermediary physiology of cyanobacteria has become important for the sustainable production of biofuels and commodity compounds from CO_2_ and sunlight by “designer microbes.” The chemical commodity product L-lactic acid can be synthesized in one step from a key intermediary metabolite of these organisms, pyruvate, catalyzed by a lactate dehydrogenase. Synthetic biology engineering to make “designer microbes” includes the introduction and overexpression of the product-forming biochemical pathway. For further optimization of product formation, modifications in the surrounding biochemical network of intermediary metabolism have to be made.

**Results:**

To improve light-driven L-lactic acid production from CO_2,_ we explored several metabolic engineering design principles, using a previously engineered L-lactic acid producing mutant strain of *Synechocystis* sp. PCC6803 as the benchmark. These strategies included: (i) increasing the expression level of the relevant product-forming enzyme, lactate dehydrogenase (LDH), for example, via expression from a replicative plasmid; (ii) co-expression of a heterologous pyruvate kinase to increase the flux towards pyruvate; and (iii) knockdown of phosphoenolpyruvate carboxylase to decrease the flux through a competing pathway (from phosphoenolpyruvate to oxaloacetate). In addition, we tested selected lactate dehydrogenases, some of which were further optimized through site-directed mutagenesis to improve the enzyme’s affinity for the co-factor nicotinamide adenine dinucleotide phosphate (NADPH). The carbon partitioning between biomass and lactic acid was increased from about 5% to over 50% by strain optimization.

**Conclusion:**

An efficient photosynthetic microbial cell factory will display a high rate and extent of conversion of substrate (CO_2_) into product (here: L-lactic acid). In the existing CO_2_-based cyanobacterial cell factories that have been described in the literature, by far most of the control over product formation resides in the genetically introduced fermentative pathway. Here we show that a strong promoter, in combination with increased gene expression, can take away a significant part of the control of this step in lactic acid production from CO_2_. Under these premises, modulation of the intracellular precursor, pyruvate, can significantly increase productivity. Additionally, production enhancement is achieved by protein engineering to increase co-factor specificity of the heterologously expressed LDH.

## Background

The growing world population and the accompanying increasing demand for materials drive our need for more sustainable production processes for biofuels and chemical commodities. Whereas microbes commonly used for fermentation processes, such as *Saccharomyces cerevisiae* and *Escherichia coli,* need sugars as their carbon source for production, cyanobacteria can directly convert CO_2_ plus the energy from sunlight directly into product [1,2]. The independence of plant-derived sugar makes a cyanobacterial cell factory more efficient and sustainable. Cyanobacteria, like *Synechocystis* sp. PCC6803 (hereafter, *Synechocystis*), can be metabolically engineered to synthesize a variety of chemical commodities, including ethanol, hydrogen, ethylene, 2,3-butanediol, alka(e)nes, isobutanol, and lactic acid [3-11]. Insertion of the appropriate (set of) heterologous enzyme(s) can divert the metabolic flux originating from CO_2_ from biomass to a product of interest.

Lactic acid, which is employed in the food and pharmaceutical industries and as a building block for biodegradable polymers [12], can be produced in *Synechocystis* after heterologous expression of a lactate dehydrogenase (LDH) [9,10,13]. In a previous study, cyanobacterial production of L-lactic acid was shown to proportionally increase with elevated expression of the L-*ldh* originating from *Lactococcus lactis*, essentially showing that the heterologous enzyme holds control over the metabolic flux towards the product [14]. Similarly, a high control by the heterologously expressed enzymes in cyanobacterial production strains was observed, for example, for ethylene [15], ethanol [16], and alka(e)nes [7].

**Figure 1:**
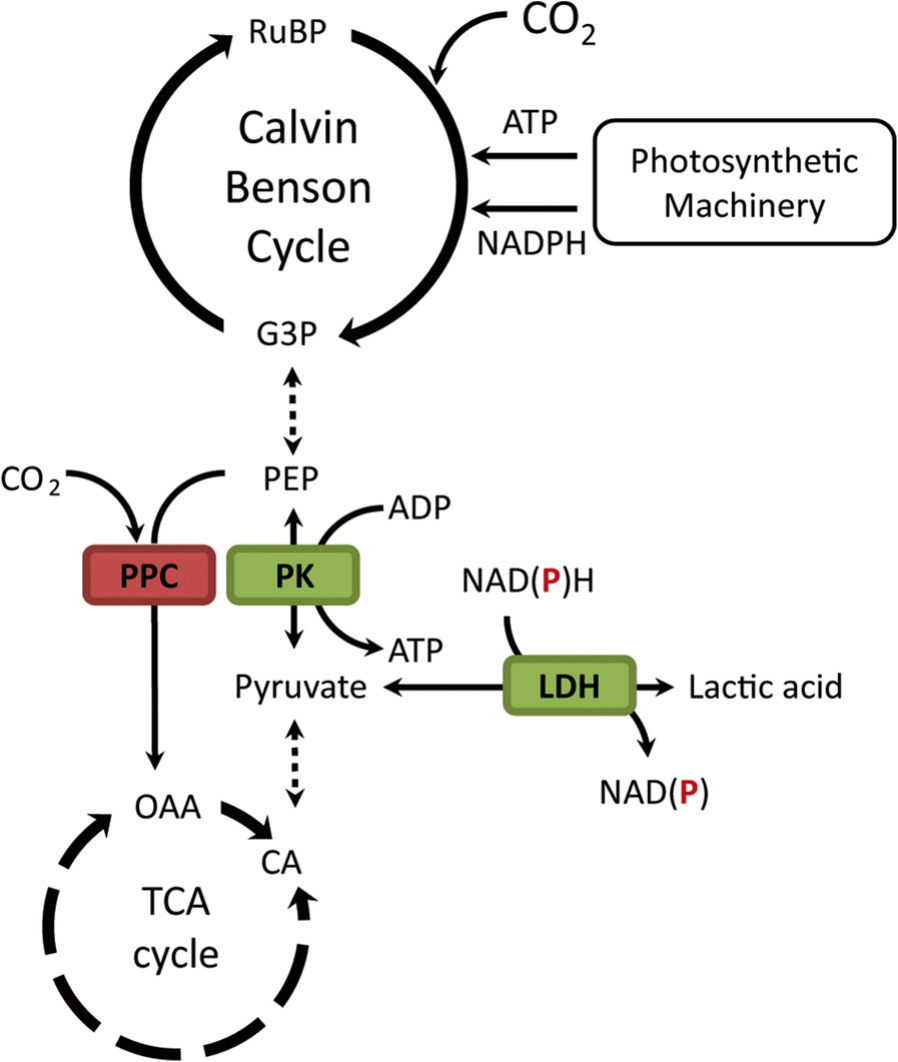
**Schematic representation of the (modified) central carbon metabolism of *Synechocystis *sp. PCC6803.** The heterologous overexpression of a lactate dehydrogenase (LDH) allows direct conversion of CO_2_ into lactic acid. Overexpression of pyruvate kinase (PK) and the partial knockout of phosphoenolpyruvate carboxylase (PPC) lead to an increase in lactic acid flux.

Here, we report the results of different strategies to further increase the production of L-lactic acid in *Synechocystis*. First, the level of expression of L-*ldh* of *L. lactis* further increased by gene cassette multiplication and the use of a self-replicating plasmid [1]. Accordingly, a protein level was reached, sufficient to divert the control over the metabolic flux towards lactic acid away from the LDH to other parts of the metabolic network. Next, we increased the metabolic flux towards lactic acid even further by targeting upstream pathways that affect the concentration of its direct metabolic precursor, pyruvate. Significantly, we show that modification of the step building up pyruvate (that is*,* pyruvate kinase, PK) and the step consuming phosphoenolpyruvate (phosphoenolpyruvate carboxylase, PPC, see Figure 1) increases lactic acid production. Additionally, we tested selected LDH enzymes with improved enzymatic properties and show that a modified LDH, with increased affinity for nicotinamide adenine dinucleotide phosphate (NADPH), originating from *Bacillus subtilis *[17], increases the lactic acid productivity.

## Results

### Increasing the gene dosage of _*Ll*_*ldh* in *Synechocystis*

CO_2_-based lactic acid production by engineered cyanobacteria can be achieved by the heterologous expression of an LDH enzyme, thereby “tapping” from the central carbon metabolism at the point of pyruvate (Figure 1), with lactic acid formation as the result [9,10,13]. In earlier studies we have shown that increased LDH activity (achieved by the overexpression of L-*ldh* of *L. lactis* (_*Ll*_*ldh*)) almost proportionally increases the production rate of L-lactic acid in *Synechocystis *[14]. This motivated us to attempt to achieve an even further increase in lactic acid productivity by expressing more LDH enzyme. The benchmark strain SAA023 (P*trc1::*_*Ll*_*ldh*_*co*_) harbors one copy of the codon optimized version of _*Ll*_*ldh,* fused to a P*trc* promoter and integrated in the genome of *Synechocystis* at the neutral docking site *slr0168 *[14]. Next we constructed strain SAA026 (SAA023 plus _*Ll*_*ldh*_*na*_), which holds an additional copy of the native version of the same gene, likewise fused to P*trc*, which essentially doubles the _*Ll*_*ldh* gene dosage. To increase the _*Ll*_*ldh* expression further, SAW035 (pDF_LDH) was constructed. SAW035 is the *Synechocystis* wild type equipped with pDF_LDH, which contains the P*trc2*-driven codon optimized version of _*Ll*_*ldh* plus the origin of replication of plasmid RSF1010 [15]. Presumably, this plasmid is present in higher copy number than the resident chromosome of *Synechocystis*[1]. The promoters P*trc* and P*trc2* essentially give rise to the same expression level and differ only with respect to the presence of one or two O1 *lac* operator sequences, respectively, which are only of importance in cases in which regulation via the LacI repressor is desired [18]. SAW039 (SAA023 plus pDF_LDH) combines the single _*Ll*_*ldh* copy on the genome with the pDF_LDH plasmid, thereby increasing the expression even further. Table 1 summarizes the strains used in this study and their respective genotypes.

**Table 1:** Strains used in this study

**Name**	**Genotype**	**Additional information**
Wild type	Wild-type *Synechocystis*	D. Bhaya (Stanford)
SAA005	P*psbA2::psbA2::*_*Ll*_*ldh*_*na*_	[14]
SAA024	P*rnpB::*_*Ll*_*ldh*_*na*_	[14]
SAA025	P*trc1::*_*Ll*_*ldh*_*na*_	[14]; P*trc1* is one *trc* promoter with one operator sequence [18]; native version of *L. lactis ldh*
SAA023	P*trc1::*_*Ll*_*ldh*_*co*_	[14]; codon optimized version of *L. lactis ldh*
SAW001	P*trc2::*_*Ll*_*ldh*_*co*_	P*trc2* is one *trc* promoter flanked by the operator sequence [18]
SAA026	P*trc1::*_*Ll*_*ldh*_*co*_*::ldh*_*na*_	Double *ldh* gene dosage on genome
SAW035	pDF_LDH	Expression from plasmid
SAW039	P*trc1::*_*Ll*_*ldh*_*co*_*,* pDF_LDH	Combined expression from genome and plasmid
SYW001	P*trc2::*_*Syn*_*pk*_*co*_*::*P*trc2::*_*Ll*_*ldh*_*co*_	*pk* from *Synechocystis*
SYW003	P*trc2::*_*Ef*_*pk*_*co*_*::*P*trc2::*_*Ll*_*ldh*_*co*_	*pk* from *E. faecalis*
SYW004	P*trc2::*_*Ec*_*pk*_*co*_*::*P*trc2::*_*Ll*_*ldh*_*co*_	*pk* from *E. coli*
SYW005	P*trc2::*_*Ll*_*pk*_*co*_*::*P*trc2::*_*Ll*_*ldh*_*co*_	*pk* from *L. lactis*
SAW041	P*trc2::*_*Ef*_*pk*_*co*_*::*P*trc2::*_*Ll*_*ldh*_*co*_*,* pDF_LDH	SYW003 additionally carrying pDF_LDH
SAW033	P*trc1::*_*Ll*_*ldh*_*co*_*, partialΔ::ppc*	Partially (stable) segregated strain of the *ppc* knockout attempt
SAA015	P*trc1::*_*Bs*_*ldh*_*na*_	[10]; native version of *ldh* from *B. subtilis*
SAA027	P*trc1::*_*Bs*_*ldh*_*co*_*WT*	Codon optimized version of *ldh* from *B. subtilis*
SAA028	P*trc1::*_*Bs*_*ldh*_*co*_*V38R*	V38R mutant of the codon optimized *ldh* from *B. subtilis*
SAA029	P*trc1::*_*Cg*_*ldh*_*co*_*WT*	Codon optimized *ldh* from *C. gunnari*
SAA030	P*trc1::*_*Cg*_*ldh*_*co*_*V53R, E232M*	V53R and E232M mutant of the codon optimized *ldh* from *C. gunnari*
SAV001	P*trc1::*_*Ll*_*ldh*_*co*_*L39R*	L39R mutant of the codon optimized *ldh* from *L. lactis*
SAA035	P*trc1::*_*Po*_*ldh*_*co*_*I29R*	I29R mutant of the codon optimized *ldh* from *P. ovale*

**Figure 2:**
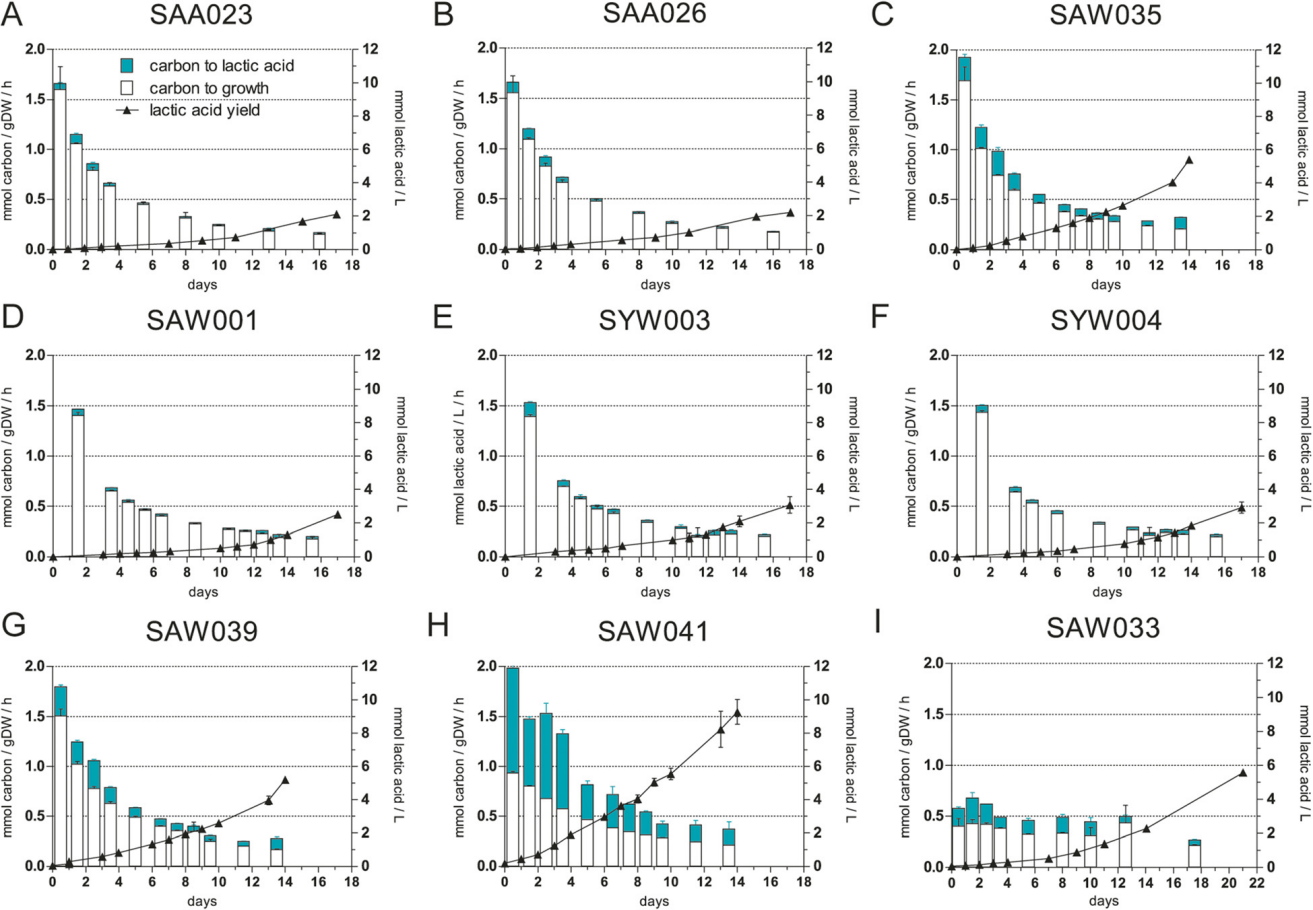
**Growth and lactic acid production in *Synechocystis *mutant strains. (A-I)** Fixed carbon partitioned into the biomass and into the product lactic acid (expressed in mmol carbon/gDW/h) at respective time intervals is shown on the left ordinate for different mutant strains expressing the LDH of *L. lactis*. Lactic acid production yield is specified by the right ordinate in mmol lactic acid/L. Error bars represent the SD of biological replicates (n = 3), except for SAW001, SYW003, and SYW004 where n = 2; if error bars are not visible, they are smaller than the data point symbol. Strain numbers are indicated above each panel, and the corresponding genotypes are given in Table 1.

A comparison of the performance of SAA026, SAW035, and SAW039 with the benchmark strain SAA023 [14] showed the largest difference within the first week of culturing in batch (Figure 2A, B, C, and G). It should be noted that production rates (metabolite fluxes) often change gradually during the course of the experiment. Various factors can limit growth and/or production in a batch culture, such as nutrient and light availability and downstream effects thereof. Consequently, the rate data presented here were derived from equivalent growth phases, namely the late-exponential phase (Additional file 1: Figure S1). However, final titers qualify as a valuable measure for overall performance of a specific mutant strain over the whole course of the experiment.

SAA026 showed roughly double the lactic acid production rate (0.0176 ± 0.0008 mmol lactic acid/gDW/h) compared to 0.0094 ± 0.0006 mmol lactic acid/gDW/h of our benchmark strain SAA023 in the late-exponential growth phase. Within one week SAA026 produced 0.56 ± 0.04, whereas SAA023 only produced 0.38 ± 0.03 mmol/L lactic acid (Figure 2A and B). SAW035 and SAW039 show higher production rates of 0.0536 ± 0.0043 and 0.0526 ± 0.0040 mmol lactic acid/gDW/h, respectively, and a buildup of 1.58 ± 0.01 and 1.61 ± 0.09 mmol/L lactic acid within one week (Figure 2C and G). The growth rate and biomass yield of the mutant strain SAA026, carrying two copies of the _*Ll*_*ldh* gene, appeared not to be affected, whereas the strains carrying the _*Ll*_*ldh* gene also on a plasmid (SAW035 and SAW039) showed mild growth retardation (Additional file 1: Figure S1) and signs of a bleaching-like phenotype.

Carbon partitioning ratios describe how much of the fixed CO_2_ is channeled into product over that used for the buildup of biomass. This parameter allows one to compare and evaluate mutant strains as “cell factory catalysts” [14,19]. Total carbon fixation is defined as carbon fixed into the product, lactic acid, plus carbon fixed into biomass. Averaging the carbon partitioning into lactic acid of the first week of culturing (that is, before perceptible carbon limitation occurs in the culture) shows a partitioning ratio of 5.4 ± 2.3%, 7.3 ± 2.0%, 17.8 ± 4.4%, and 18.7 ± 4.2% for SAA023, SAA026, SAW035, and SAW039, respectively (Table 2).

**Table 2:** Increased carbon partitioning into lactic acid with increasing LDH expression

**Strain**	**SAA023**	**SAA026**	**SAW035**	**SAW039**
Carbon partitioning [%]	5.4 ± 2.3	7.3 ± 2.0	17.8 ± 4.4	18.7 ± 4.2

Partitioning of the incoming CO_2_ over lactic acid and biomass, and expressed as the percentage of the total carbon fixation ending up in lactic acid. Values represent the average and SD derived from the first week of growth (compare Figure 2). Data has been derived from triplicate cultures.

**Figure 3:**
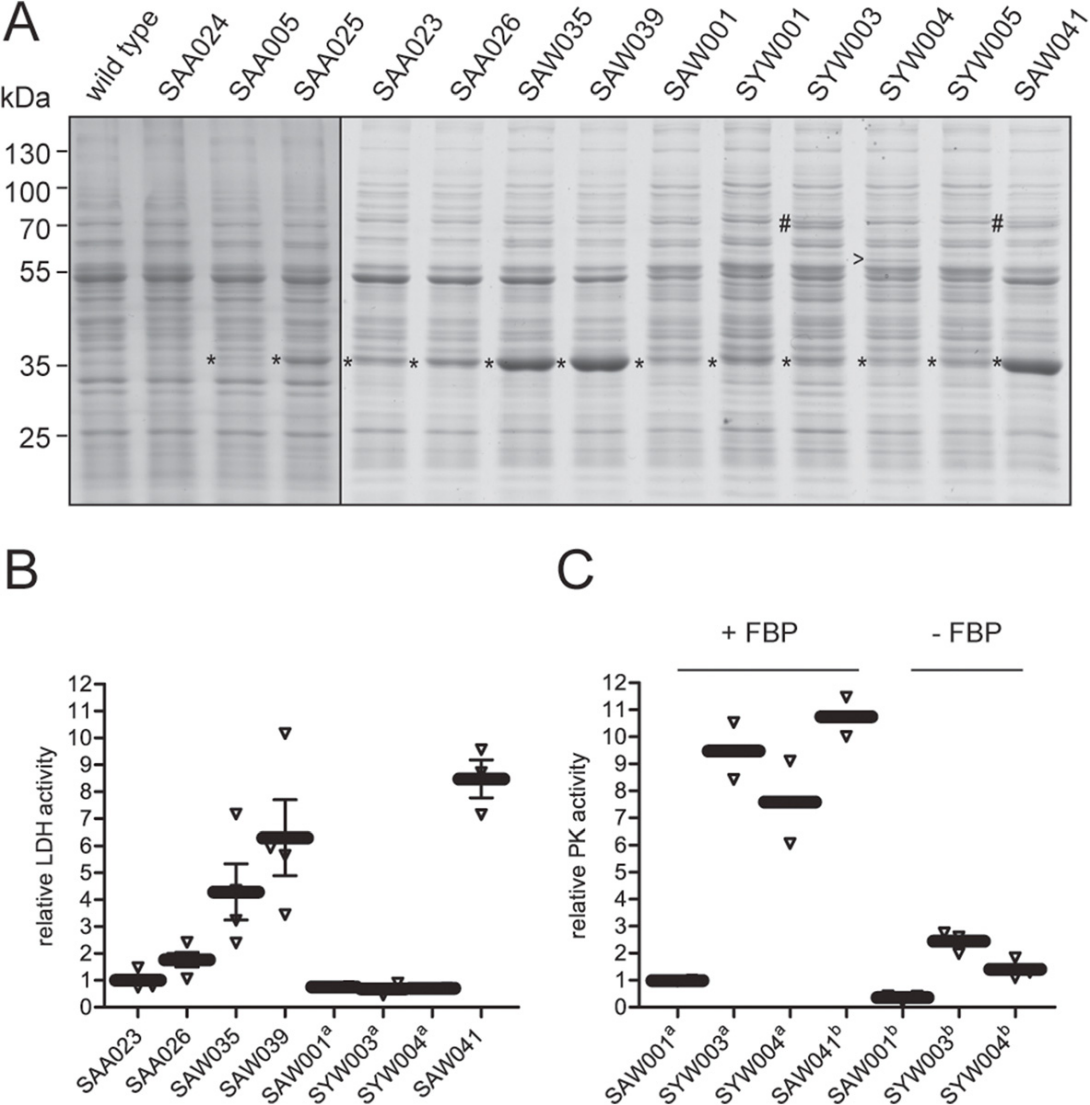
**Expression and activity of LDH and PK in mutant strains of *Synechocystis*. (A)** Coomassie Brilliant Blue (CBB)-stained gel of cell-free extracts of *Synechocystis* mutant strains showing overexpression of the LDH of *L. lactis* (*), the PK of *E. faecalis* (#), and the PK of *E. coli* (>). The vertical line indicates a composite gel. Lanes are aligned according to the pattern of the wild-type proteins visible in the cell-free extract. **(B)** Enzymatic activity of LDH in cell-free extracts of relevant mutant strains, showing the relative increase with respect to the benchmark strain SAA023. **(C)** Enzymatic activity of PK in cell-free extracts with (+) and without (-) fructose-1,6-bisphosphate (FBP), showing the activity relative to the reference strain SAW001. Error bars represent the SD of biological replicates (n = 3); if error bars are not visible, they are smaller than the symbol of the data point; ^a^in (B) and (C) the values represent the mean of biological duplicates, and ^b^in (C) the mean of technical triplicates. In both (B) and (C) the black bar represents the mean, and the open triangles represent the biological, or technical replicates, respectively.

**Figure 4:**
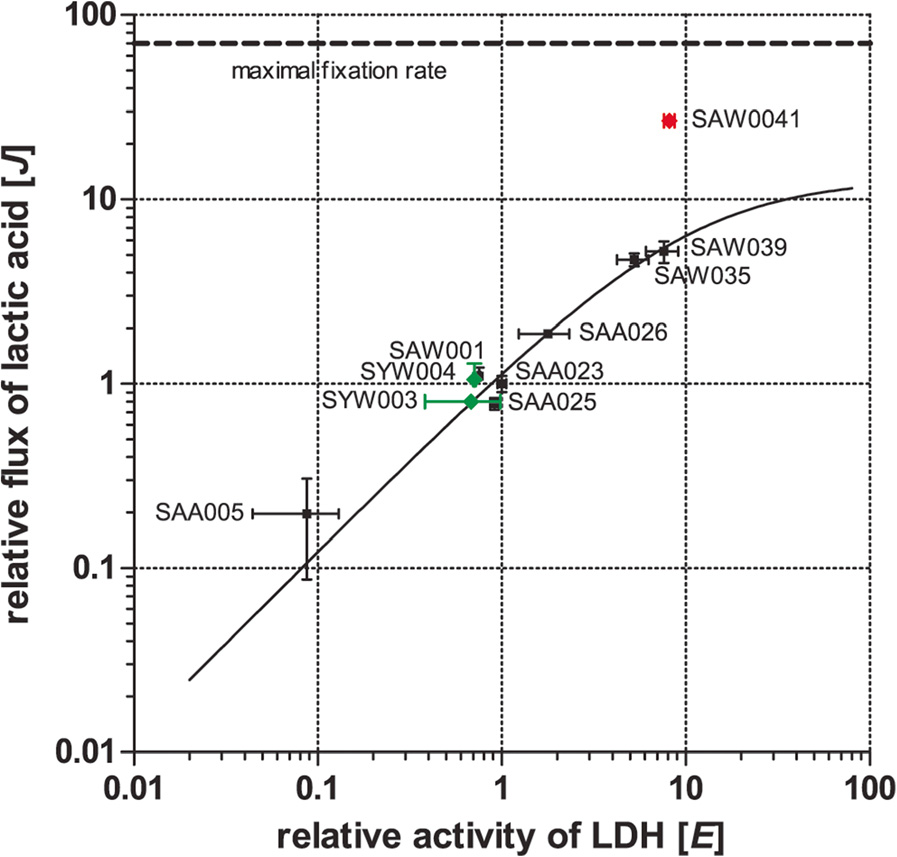
**Analysis of flux control over lactic acid formation.** Double logarithmic plot relating relative LDH enzyme activity (abscissa) with relative flux towards the final product lactic acid (ordinate). Mutant strains that contain LDH as the only exogenous gene (black squares) were used to generate the fit (black solid line) and were used to calculate control coefficients. Data for the PK co-expressing strains with low amounts of LDH expression, SYW003 and SYW004, are shown with green diamonds. SAW041, the strain with PK co-expression with high amounts of LDH expression, is shown with a red diamond. Data for mutant strains SAA005 and SAA025 were taken from [14]. Error bars represent the SD of biological replicates (n = 3), except for SAW001, SYW003, and SYW004 where n = 2; if error bars are not visible, they are smaller than the data point symbol.

The observed increased lactic acid productivity correlates well with the observed increase in enzyme expression (Figure 3A) and the elevated LDH activity determined in cell-free extracts (Figure 3B). Relative to the benchmark strain SAA023, the enzymatic activity of LDH from the overexpression strains was increased by 1.82 ± 0.42, 7.17 ± 1.07, and 10.16 ± 1.66 fold for SAA026, SAW035, and SAW039, respectively (Figure 3B). Correlating the relative LDH activity with the relative production rate allows one to determine the control coefficient for the enzyme in question [20]. In view of the strong dependence of the production rate on growth phase, care must be taken to estimate both characteristics at the same phase of growth. To improve the accuracy of the estimate of this control coefficient, we added data obtained with two comparable mutant strains (SAA005 (P*psbA2-*driven native _*Ll*_*ldh*) and SAA025 (P*trc1-*driven native _*Ll*_*ldh*), compare Figure 3 and [14]) that express exactly the same LDH, but less of it than the benchmark strain SAA023. The relative LDH activity and relative production rate were first plotted on linear scales and a rectangular hyperbola was fitted through the data, based on a Michaelis-Menten-type saturation curve [20] (Additional file 1: Figure S2). This fit was used for a numerical estimate of the flux control coefficient for the LDH in the respective mutants. Figure 4 shows the same data in a double logarithmic plot. The numerical estimate of the control coefficient of LDH in the respective mutants, based on the correlation between the enzymatic activity [*E*] values and the values of the production rate (flux) [*J*] (Table 3), showed that an increase in activity resulted in an almost proportional increase in flux. This is visible from mutant strain SAA005, expressing low amounts of LDH, up to mutant strain SAA026, expressing medium amounts of LDH. However, this proportional relationship breaks down for the two mutant strains expressing the highest amounts of LDH, that is, SAW035 and SAW039 (Table 3). Hence, the introduced LDH has a control coefficient close to 1 for the strains SAA005, SAA025, SAA023, and SAA026, but in the the strains SAW035 and SAW039 it shows a significant decrease, approximating 0.5 in SAW039 (Table 3).

**Table 3:** **Analysis of the flux control coefficient of LDH in recombinant *Synechocystis *strains**

**Strain**	**Relative activity [*E*]**	**Relative flux [*J*]**	**Flux control coefficient [*C***_***J***_^***E***^**]**
Wild type	0	0	n.a.
SAA024*	0	0	n.a.
SAA005*	0.09 ± 0.04	0.20 ± 0.11	0.99
SAA025*	0.91 ± 0.05	0.78 ± 0.06	0.92
SAW001	0.75 ± 0.01	1.09 ± 0.13	0.93
SAA023	1.00 ± 0.06	1.00 ± 0.10	0.91
SAA026	1.78 ± 0.55	1.87 ± 0.08	0.84
SAW035	5.25 ± 1.03	4.69 ± 0.37	0.59
SAW039	7.59 ± 1.51	5.21 ± 0.70	0.50
SYW003	0.68 ± 0.30	0.80 ± 0.07	n.a.
SYW004	0.71 ± 0.01	1.05 ± 0.23	n.a.
SAW041	8.15 ± 0.53	26.67 ± 2.16	n.a.

Enzyme activity and lactate production rate are expressed relative to the benchmark strain SAA023. Flux control coefficient values are determined from the slope of the tangent to the fitted curve in Figure 4, for the respective relative activity values. Data for SAA005, SAA024, and SAA025 (*) were taken from [14]. Error ranges represent the SD of biological replicates (n = 3), except for SAW001, SYW003, and SYW004 where n = 2; n.a. not applicable.

In conclusion, although complete saturation of the rate of lactic acid production with respect to LDH activity was not achieved, the control of the LDH activity over the flux towards the product could be lowered significantly. This suggests that introducing modifications elsewhere in the metabolic network, rather than further elevating the expression level of the introduced exogenous (fermentative) pathway, may turn out to be more effective in further increasing productivity [21]. Below we present the results of such an approach via altering the levels of pyruvate kinase (PK) and phosphoenolpyruvate (PEP) carboxylase (PPC).

### Co-expression of a pyruvate kinase increases lactic acid production

The intracellular level of pyruvate, the immediate precursor for lactic acid, in *Synechocystis* cells is expected to be well below the K_M_ of the L-LDH of *L. lactis *[22,23]. Thus, we set out to further improve lactic acid productivity by attempting to increase the flux towards pyruvate. In the step prior to the LDH-catalyzed reaction, pyruvate kinase (PK) converts PEP into pyruvate (Figure 1), making PK an attractive target for overexpression. Heterologous PK expression has previously been successfully applied to increase the flux to ethanol in genetically modified *E. coli *[24]. Furthermore, heterologous overexpression of an upstream enzyme, ribulose-1,5-bisphosphate-carboxylase/oxygenase, has been shown to benefit the production of isobutyraldehyde in *Synechococcus elongatus* PCC7942 [8].

We chose five different PK enzymes for this overexpression, namely those of *Synechocystis *[25], *Bacillus subtilis *[26], *Enterococcus faecalis*, *E. coli *[27], and *L. lactis *[28]. The annotation of the *Synechocystis* genome revealed two pyruvate kinases, *sll0587* and *sll1275*, but only the latter, encoding a PK-A (that is, an AMP-activated PK), is expressed [25]. The five corresponding pyruvate kinase genes (*pk*) were codon optimized for *Synechocystis* as described in [6] and fused to P*trc2*. Overexpression of PK was tested in the background of SAW001 (a SAA023-like strain with P*trc2* replacing P*trc1*), where a single copy of _*Ll*_*ldh* is driven by P*trc2*. As expected, the lactic acid productivity, as well as the expression level of LDH of SAW001, is essentially identical to that of SAA023 (Figure 2A and D, and Figure 3). The *Synechocystis* strains carrying the _*Ll*_*ldh* and any one of the five *pk* genes were named SYW001 to SYW005 (Table 1). Despite numerous attempts, we were not able to create a *Synechocystis* mutant strain carrying the *pk* of *B. subtilis* (that is, the tentative SYW002 strain).

For two strains, SYW001 (SAW001 plus _*Syn*_*pk*) and SYW005 (SAW001 plus _*Ll*_*pk*), carrying the genes coding for PK from *Synechocystis* (*sll1275*) and *L. lactis*, respectively, no additional effect on lactic acid productivity was observed as compared to SAW001. Furthermore, expression of the respective PK was not detected on Coomassie Brilliant Blue (CBB)-stained sodium dodecyl sulfate polyacrylamide gel electrophoresis (SDS-PAGE) (Figure 3A), and PK activity assays did not show significant activity over the background (data not shown). In contrast, SYW003 (SAW001 plus _*Ef*_*pk*) and SYW004 (SAW001 plus _*Ec*_*pk*) did show expression of the respective PK on SDS-PAGE (Figure 3A) as well as PK activity in the cell-free extract (Figure 3C). The employed PK-F of *E. coli* is an FBP-activated PK [27]. The activity assays confirmed the FBP dependency of the selected PK-F enzymes (Figure 3C). The LDH activity in these heterologous PK-containing mutants was identical to that of the reference strain SAW001 (Figure 3B). Comparison of SYW003 and SYW004 with SAW001 shows a small positive effect of PK expression on the rates and titers of lactic acid production (Figure 2E and F), resulting also in slightly higher carbon partitioning ratios during most of the time of culturing in the first week (Table 4) and moderately higher volumetric production rates (Additional file 1: Figure S1). From these results we can conclude that the co-overexpression of a functional PK in the background of a single copy of _*Ll*_*ldh* does not change the production rate profoundly. This is consistent with the results of the control analysis that showed that the major part of the control over the lactic acid flux in these recombinant strains is held by the LDH (Table 3).

**Table 4:** Effect of co-expression of PK on carbon partitioning into lactic acid

**Strain**	**SAW001**	**SYW003**	**SYW004**	**SAW041**
Carbon partitioning [%]	6.3 ± 1.9	7.2 ± 2.1	5.5 ± 0.7	50.0 ± 5.9

Partitioning of fixed CO_2_ into lactic acid, expressed as percentage of the total carbon fixation rate (the flux of carbon into product over biomass). Values represent the average and SD derived from the first week of growth (compare Figure 2). Data is derived from replicate cultures.

To test the effect of PK overexpression in a strain where the LDH holds significantly less control over the lactic acid flux, we introduced the pDF_LDH plasmid into SYW003, resulting in mutant strain SAW041 (SYW003 plus pDF_LDH). Strikingly, SAW041 showed a high lactic acid production rate and final titer (Figure 2H and Additional file 1: Figure S1H). SAW041 can be directly compared with SAW039, which has comparable expression and activity levels of the L-LDH of *L. lactis* (Figure 3A and B) but no additional PK. In two weeks the lactic acid concentration increased from 5.20 ± 0.17 in SAW039 to 9.29 ± 0.74 mmol/L lactic acid in SAW041, but the additional PK enzyme also leads to notable growth impairment (Additional file 1: Figure S1). The combination of a higher volumetric production rate and a decreased growth rate resulted in an increased partitioning ratio for SAW041, with an average of 50.0 ± 5.9% during the first week of culturing (Table 4). The production rate at the late-exponential growth phase increased almost fivefold from 0.0526 ± 0.0040 in SAW039 to 0.2512 ± 0.0203 mmol lactic acid/gDW/h in SAW041 (Figure 2G and H). By plotting the characteristics of SAW041 in Figure 4, one can see that the loss of control of LDH in SAW039 can be successfully used to increase lactic acid production at constant levels of LDH activity, by the additional expression of a functional PK.

### Knockdown of PEP carboxylase increases lactic acid production

Rational metabolic engineering for biosynthesis typically also makes use of genetic modifications in the form of knockouts (for example, of competing pathways), which can increase product formation [29]. To further optimize the pathway towards lactic acid formation, we also targeted an upstream branching point, that is, a reaction that competes with the conversion of PEP to pyruvate, namely the PEP carboxylase (PPC) step (Figure 1). As a significant flux of carbon could “bypass” pyruvate via formation of oxalic acid as a tricarboxylic acid (TCA) cycle intermediate [30-32], we set out to make a PPC knockout mutant, via targeting of the *ppc* gene by double homologous recombination with an antibiotic resistance gene. As to be expected for an essential gene, and also previously observed in *Synechococcus elongatus* PCC7942 [33], we could not establish complete segregation of the resulting transformant. However, by application of low antibiotic pressure (5 μg/mL), we could stably maintain and subsequently test the partially segregated strains. These strains were highly attenuated in growth rate, which is likely caused by the lower expression of the PPC enzyme. We introduced this *ppc* modification in the background of SAA023, which resulted in the lactic acid producing strain SAW033 (SAA023 with a partial *ppc* deletion). The growth rate of SAW033 was severely impaired (Additional file 1: Figure S1I). Interestingly, lactic acid production rates in this strain are comparable to the rates found in the strains strongly overexpressing the L-LDH of *L. lactis* (SAW035 and SAW039) (Figure 2I). This resulted in an average partitioning ratio for lactic acid of 30.3 ± 5.0% for the first week of culturing of SAW033. Furthermore, in this strain relatively high volumetric production rates (0.04 mmol lactic acid/L/h, compare Additional file 1: Figure S1I) and final titers of 2.28 ± 0.11 mmol/L lactic acid were reached (Figure 2I). The total rate of carbon fixation of this strain was, however, diminished (Figure 2I). In conclusion, the partial knockout (that is, a knockdown) of *ppc* clearly affects the physiology of the cells in a severe manner, which results in a slow-growth phenotype, but also in an increased partitioning of carbon into lactic acid, as compared to the benchmark strain SAA023.

### Testing selected lactate dehydrogenases

Increased channeling of the carbon flux towards a preferred end product like lactic acid can also be modulated by engineering the kinetic properties of the selected heterologous fermentative enzyme [6,19]. We therefore searched the literature for L-LDH enzymes with low K_M_ values for pyruvate and high turnover numbers (or k_cat_ values). Based on these criteria, we consulted the enzyme database BRENDA [34] and selected three alternative LDH enzymes: an LDH from *Champsocephalus gunnari* (a mackerel icefish) [35]*,* an LDH from *Plasmodium ovale* (belonging to the parasitic protozoa) [36], and the LDH from *Bacillus subtilis* (a Gram-positive bacterium). Of the latter we have previously tested a non-codon optimized version of the gene in *Synechocystis *[10]*.* The reported kinetics for these three enzymes appeared superior to the kinetics of LDH of *L. lactis *[23]. In the LDH of *C. gunnari* we introduced a mutation (E232M) that has previously been identified to lower the K_M_ for pyruvate from 0.5 to 0.3 mmol/L (at 20°C) in a comparative study of enzyme kinetics of LDHs from different Antarctic fish [37].

**Figure 5:**
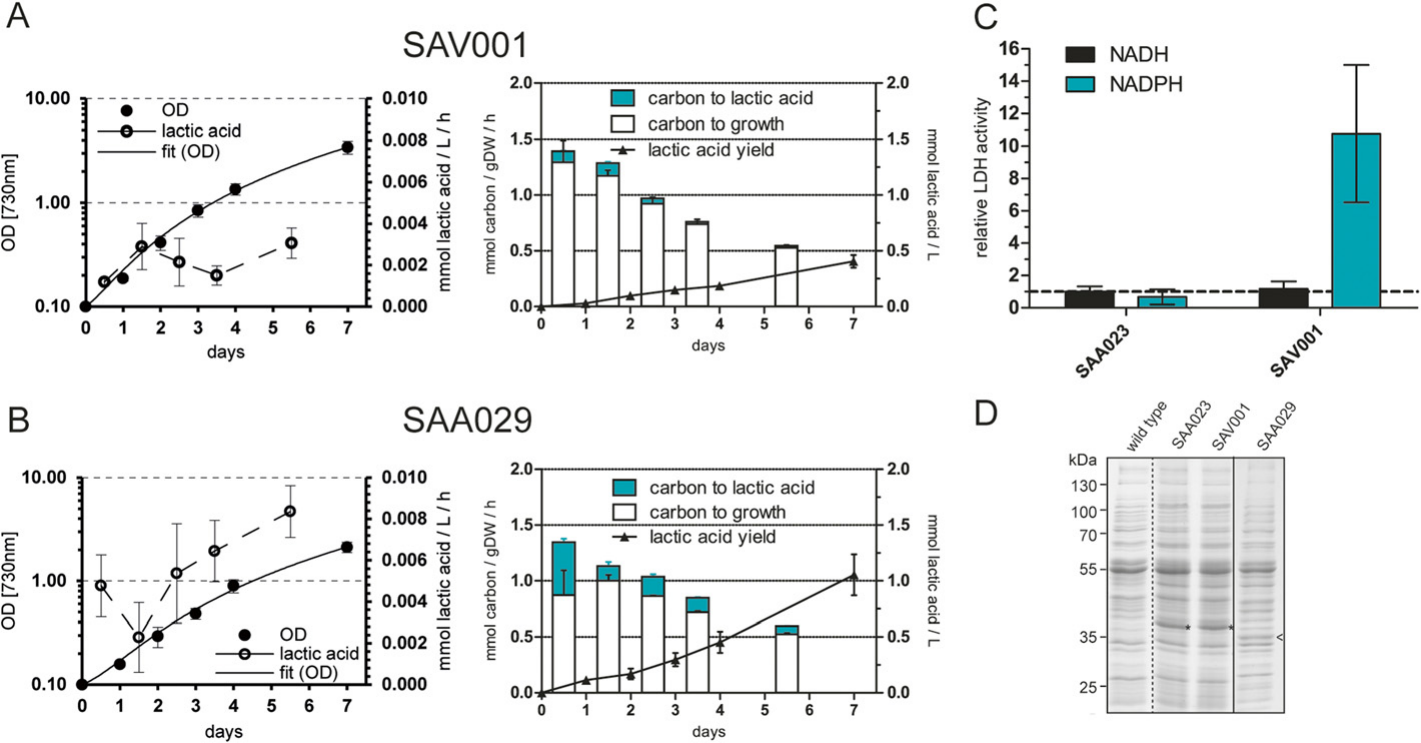
**Growth and lactic acid production in *Synechocystis *mutant strains expressing alternative LDH enzymes. (A, B)** Growth curves and (volumetric) rate of production (left chart); L-lactic acid yield and partitioning (right chart) for **(A)** SAV001, expressing the *Ll*LDH_L39R_ and **(B)** SAA029, expressing the *Cg*LDH_WT_, and **(C)** Enzymatic activity of cell-free extracts of SAV001 towards NADH and NADPH, relative to the NADH-dependent activity of SAA023. Error bars represent the SD of biological replicates (n = 3); if error bars are not visible, they are smaller than the data point symbol. **(D)** CBB-stained gel with corresponding cell-free extracts, indicating the overexpression of the unmodified and *Ll*LDH_L39R_ in SAA023 and SAV001, respectively (*), and the *Cg*LDH_WT_ in SAA029 (<). The solid vertical line indicates a composite gel. Lanes are aligned according to the pattern of the wild-type proteins visible in the cell-free extract. The stippled vertical line separates wild type from the mutant strains’ cell-free extracts.

**Figure 6:**
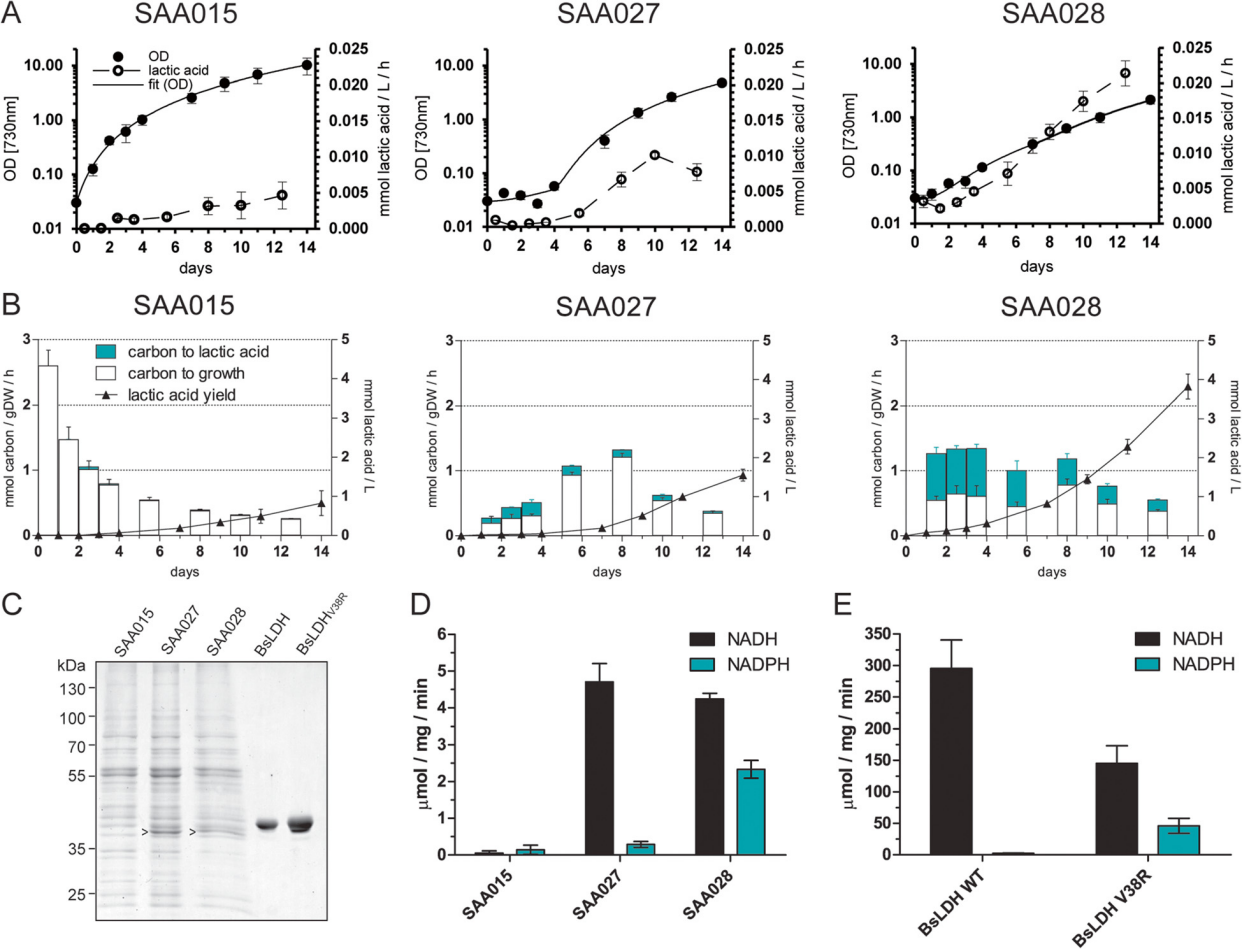
**Growth and lactic acid production of *Synechocystis *strains overexpressing the L-LDH**_**WT **_**and the L-LDH**_**V38R **_**of *B. subtilis*. (A)** Growth curves and (volumetric) lactic acid rates over time of SAA015 (expressing the non-codon optimized *ldh*), SAA027 (expressing a codon optimized *ldh*), and SAA028 (expressing the V38R-mutated version of the codon optimized *ldh* of *B. subtilis*). **(B)** Fixed carbon partitioned over biomass and the product lactic acid, at the subsequent respective time intervals (left ordinate). Lactic acid production yields (filled triangles) are indicated on the right ordinate. **(C)** CBB-stained gel with corresponding cell-free extracts and the His-tagged purified proteins. **(D)** Enzymatic activity of cell-free extracts of SAA015, SAA027, and SAA028. **(E)** Enzymatic activity of the purified enzymes, *B. subtilis* LDH_WT_ and LDH_V38R_. Error bars represent the SD of biological replicates (n = 3).

The three constructed strains were tested for growth and lactic acid production, and compared to the benchmark strain SAA023. All genes were codon optimized and placed under the control of P*trc*, and inserted as a single copy into the genome of *Synechocystis.* The mutant strain carrying the *ldh* of *C. gunnari* (SAA029) performed slightly better than SAA023, but suffered from a clear growth defect (Figure 5B). After one week of growth, extracellular lactic acid had accumulated to 1.05 ± 0.18 mmol/L (Figure 5B), whereas the benchmark strain SAA023 only produced 0.38 ± 0.03 mmol/L lactic acid (Figure 2A). Analyzing the soluble lysate of this strain on a CBB-stained SDS-PAGE gel showed that in SAA029 the LDH of *C. gunnari* is expressed to approximately similar levels as the LDH of *L. lactis* in SAA023 (Figure 5D), suggesting that the LDH of *C. gunnari* has indeed more favorable kinetic properties. In contrast, the strain carrying a mutated version of the LDH of *P. ovale* (LDH_I29R_ in strain SAA035) exhibited normal growth but no lactic acid production. Furthermore, SAA035 did not show detectable LDH activity in the cell-free extract or a protein band upon CBB-stained SDS-PAGE (data not shown), suggesting lack of expression of this enzyme. The mutant strain carrying the codon optimized wild-type version of the *B. subtilis* LDH (SAA027 expressing _*Bs*_*ldh*_*co*_) shows significantly higher productivity than the benchmark strain SAA023, but hampered growth (Figure 6A). In contrast to earlier findings for the *ldh* of *L. lactis* in *Synechocystis*[14], codon optimization significantly increased expression levels for the *B. subtilis ldh*. Furthermore, SAA027 exhibited much higher production rates (Figure 6B) and higher LDH activity (Figure 6D) than SAA015, in which the native *B. subtilis ldh* (_*Bs*_*ldh*_*na*_) is employed [10]. Figure 6C shows that the LDH of SAA015 is barely expressed, whereas the codon optimized version in SAA027 shows high expression levels.

These results emphasize that successful product formation by a heterologous pathway depends on a combination of both the kinetics of the introduced enzyme (hence on the choice of the enzyme) and on successful overexpression, which may be influenced by codon usage.

### Changing the co-factor specificity of the LDH

Utilizing redox-active enzymes with different co-factor specificity has been employed previously in cyanobacteria [6,19,38]. Because electrons liberated from water in the thylakoid membrane become available for intermediary metabolism in the form of NADPH, it has been proposed that cyanobacteria possess a large pool of NADP(H), which is even more reduced than the NAD(H) pool [22,39], at least under light saturating conditions in the photoautotrophic mode of growth. An NADPH-dependent LDH enzyme would therefore presumably be optimal for lactic acid production. Unfortunately, in the literature we did not find LDH enzymes that solely depend on NADPH. Nevertheless, the *B. subtilis* LDH enzyme has been reported to be able to co-utilize NADPH, next to NADH [17] upon site-directed mutagenesis of V38. We introduced the reported V38R mutation, via a single point mutation, in the codon optimized *ldh* gene (for details see the Methods section). Indeed, the employment of this engineered enzyme in the *Synechocystis* strain SAA028, which is expressing the codon optimized gene of the V38R version of the *B. subtilis ldh,* did lead to a higher lactic acid production rate, as compared to the employment of the wild-type version of this enzyme (SAA027), as can be seen in Figure 6A and B. The accumulation of lactic acid after two weeks of growth increased from 1.56 ± 0.15 in strain SAA027 to 3.83 ± 0.32 mmol lactic acid/L in strain SAA028. As shown in Figure 6A, both mutants display a significant lag phase, followed by a lower growth rate in the exponential phase of growth, compared to SAA015 (compare Figure 6C). Furthermore, strain SAA028 shows a lower maximal growth rate than SAA027 (Figure 6A), which leads to an increased partitioning of carbon into product instead of into biomass (Figure 6B). SAA027 shows a production rate of 0.0366 ± 0.0024 mmol lactic acid/gDW/h during the late-exponential growth phase and a carbon partitioning into lactic acid of 31 ± 13% in the first week of culturing, whereas SAA028 shows a higher production rate of 0.1343 ± 0.0266 mmol lactic acid/gDW/h and a carbon partitioning into lactic acid of 55 ± 2%. Analysis of cell-free extracts *in vitro* showed that its activity with NADH did not decrease significantly, but that the activity with NADPH is significantly higher in SAA028 expressing LDH_V38R_ (Figure 6D). Likewise, the characterization of the purified enzymes *in vitro* with respect to utilization of NAD(P)H showed a similar increase, albeit its maximal turnover activity with NADH decreased (Figure 6E). Determination of the K_M_ for pyruvate resulted in a value of 4.34 ± 1.24 for the wild-type version of the *B. subtilis* LDH and 4.00 ± 0.50 mmol/L for the V38R version of the *B. subtilis* LDH, indicating no significant change (Additional file 1: Figure S3). This emphasizes that the altered rate and level of lactic acid production in SAA028 stems from the engineered increased activity with NADPH. It should be noted, however, that further sub-culturing experiments (data not shown) resulted in a reduction of lactic acid production and a (genetic) instability of the mutant strain SAA028, which prevented further (genetic) modifications. More specifically, single point mutations in the open reading frame (ORF) of the modified *B. subtilis ldh* appeared, likely affecting its expression level and/or the activity of the LDH.

A similar mutation in the NADH binding pocket was been introduced into the LDH from *P. ovale*, *L. lactis,* and *C. gunnari*, based on alignments using Clustal Omega (Additional file 1: Figure S4). The *Synechocystis* strain expressing the mutated *ldh* gene of *P. ovale* (SAA035) was discussed in the previous section. SAV001, the strain expressing the mutated version V39R of the *L. lactis ldh,* can be directly compared to the benchmark strain SAA023, which expresses the original wild-type version of the *L. lactis ldh*. The cell-free extract of SAV001 showed an approximately tenfold increase in activity with NADPH compared to SAA023 (Figure 5C). Both show similar activity for NADH (Figure 5C) and similar expression levels (Figure 5D). This indicates that the introduced point mutation resulted in the desired increase in NADPH co-utilization for this enzyme tested *in vitro* for the cell extract. However, the lactic acid titers in SAV001 (Figure 5A) were similar to the titers in SAA023 (compare Figure 2A). SAA030, the strain expressing the mutated version V53R, E232M of the *C. gunnari ldh,* was compared to strain SAA029 (which is expressing the same *ldh* but without the V53R mutation. SAA030 did not show increased lactic acid production (data not shown), as compared to SAA029, although an increased activity of the heterologous enzyme with NADPH, tested in the cell-free extract, was observed (data not shown). Moreover, strain SAA030 was genetically unstable and rapidly lost the ability to produce lactic acid, indicating that expression of the mutated version V53, E232M of the *C. gunnari ldh* imposes a significant metabolic burden on the growth of the cells.

## Discussion

In this study, we have systematically applied different strategies to increase the production of L-lactic acid in *Synechocystis.* First, we gradually raised the expression level of the L-LDH of *L. lactis* by gene multiplication and expression from a plasmid, starting from benchmark strain SAA023 [14], finally resulting in LDH levels that showed more than a tenfold increased LDH activity in strain SAW039. Although LDH activity shows a small but measurable increase from SAW035 to SAW039, the lactic acid production rate does not. Whether or not the rate of lactic acid production has reached an optimum in strain SAW039 cannot be decided yet. However, derived from the metabolic control analysis (Figure 4), we conclude that with the further overexpression of solely the L-LDH of *L. lactis*, a further large increase in flux towards lactic acid cannot be gained. The introduced fit in Figure 4 shows a relative maximum rate of lactic acid production of 12.99 ± 3.3 (Additional file 1: Figure S2). With the strategy of increasing LDH activity alone, the gain in production rate to the level of this calculated maximum could only be achieved by increasing LDH expression more than tenfold (Figure 4). However, the protein level of LDH enzyme in the soluble fraction of, for example, strain SAW039 (Figure 3A) is already high compared to the native proteins visible, suggesting that a many-fold increase in LDH activity can no longer be achieved. Moreover, very high expression levels of the introduced heterologous enzyme may give rise to a decrease, rather than an increase, in the rate of product formation because of the associated burden that the cells may face from its metabolic load [40]. Recently, in a strain that expressed a heterologous protein to 15% (w/w) of the total soluble protein content, no negative effect on growth rate was noted [41]. It should be noted that the strains expressing large amounts of LDH protein (SAW035 and the more productive mutant strains) did show impaired growth. The latter could be due to: (i) channeling of carbon into product instead of into biomass, which would imply that the desired cell factory has been constructed, (ii) an energy burden on the cells due to the biosynthesis of the LDH enzyme, (iii) physiological constraints that impair growth due to the introduced enzyme that, for example, utilizes the NADH/NAD redox couple, whose balance may be important for the cell, or (iv) a combination of these factors.

In principle, the flux of lactic acid can converge with the maximum CO_2_ fixation rate, if all conditions are optimal in the cell. This leads to the conclusion that the theoretical maximum rate of lactate production, equal on a carbon basis to the maximum rate of carbon fixation (the dashed line in Figure 4, which can be calculated from the reported maximum growth rate [42]), can only be reached by using different and/or additional modifications, genetic or physiological. A combined approach, that is, a further increase in LDH enzyme activity and an optimization of the intracellular conditions for LDH activity, may be required for this. For a further increase in the lactic acid flux it is therefore important to also target the precursor metabolite pyruvate (via the modification at the steps of pyruvate kinase (PK) and phosphoenolpyruvate (PEP) carboxylase (PPC)). Interestingly, the native PK activity at photoautotrophic conditions in the light is hypothesized to be down-regulated compared to the dark activity level [43]. Recent studies suggest that, in the absence of carbon limitation [31], a significant flux of carbon is channeled via PPC through oxaloacetate, malate dehydrogenase, and the malic enzyme to pyruvate, rather than forming pyruvate directly from the ATP-generating reaction catalyzed by PK [30]. Thus, the overexpression of a malic enzyme may be an additional way to boost lactic acid formation. Alternatively, the overexpression of PEP carboxykinase might increase the flux towards pyruvate [44]. Significantly, our attempts to employ a malolactic enzyme to directly convert malate into lactic acid did not lead to any stimulation of lactic acid production (data not shown).

We could show that the overexpression of PK further boosted lactic acid formation. Interestingly, the overexpressed PK affected productivity under conditions in which the LDH was highly expressed. These results confirm that at such high expression levels the LDH has lost a significant part of the control over the rate of lactic acid formation. In contrast, in the *ppc*-knockdown strain SAW033, the rate of lactic acid production is increased, even with lower LDH enzyme expression levels. This result suggests that *ppc* knockdown either more strongly increases pyruvate levels than the overexpression of PK, or that this modification changes metabolism to such an extent that it affects other factors that play a role in lactic acid formation.

Although carbon partitioning to lactate in SAW033 is quite high, growth in this mutant is severely reduced, and thus the total rate of carbon fixation for lactic acid production is also lower. The reduced carbon fixation rate in SAW033 can be partially explained by the fact that the PCC enzyme can actually fix CO_2_ and is responsible for part of the carbon fixation in *Synechocystis *[31]. Clearly, the negative effect of *ppc* knockdown on the total rate of carbon fixation rate does not outweigh the positive effect this knockdown has on the partitioning of the carbon flux to lactic acid.

We have shown that we could increase productivity by introducing a point mutation in the *B. subtilis* LDH to create a co-factor promiscuous enzyme. The observed increased productivity with the NADPH-co-utilizing LDH, as compared to the wild-type enzyme that is specific for NADH, could be due to (i) the fact that NADPH is more abundant than NADH in photosynthetic organisms [22], or could occur because (ii) the ratio of the redox couple NADPH/NADP is higher than that of NADH/NAD [22,39], or (iii) simply because now the engineered enzyme is able to use both pools: NADPH and NADH. The employment of this enzyme also resulted in a reduced growth, possibly partially due to the carbon channeled into lactic acid, but the additional NADPH consuming reaction might also have some effect on the physiology of the cells. Nonetheless, for cyanobacterial cell factories producing the relatively reduced compounds, it might help to change the co-factor affinity of reductases of which no natural NADPH-using version is available. However, as shown in this study, the effect of point mutations that change co-factor usage cannot easily be inferred from other, albeit homologous, proteins. Likewise, the expression of several enzymes, such as the anticipated expression of the *P. ovale* LDH_I29R_, did not yield the desired effect of expression, let alone stable, high-level production. The strain expressing the LDH from *C. gunnari* performed much better, but still not to the expected levels based on the enzymes’ catalytic properties. However, the natural physicochemical environment of this enzyme (residing in the cells of an Antarctic icefish) is undoubtedly rather different from the one in *Synechocystis*.

It is relevant to note that enzymes with dehydrogenase (and specifically lactate dehydrogenase) activity, converting pyruvate with the co-factor NADP(H) in a fermentative catabolic reaction, seem rare in nature. In general, NADPH-dependent reactions take part in anabolic pathways of metabolism. Interestingly, the wild-type version of the *B. subtilis* LDH is capable of oxidizing NADPH, but it shows a K_M_ that is more than 20-fold higher than that for NADH [45]. Likewise, the lactate dehydrogenase of the potato tuber shows a more than threefold higher K_M_ for NADPH than for NADH, when assayed with excess pyruvate [46]. In higher eukaryotes one does find highly NADPH-dependent reductases for hydroxypyruvate; however, these do not seem to show any affinity for pyruvate itself [34,47].

In conclusion, in this study the enzyme of interest (that is, LDH) has been overexpressed to a close-to-optimal level for the design of a cyanobacterial cell factory for lactic acid. The newly introduced metabolic pathway “pulling” on an intracellular metabolite represents the product-forming pathway, but also an important bottleneck in overall product formation. Modifications, such as overexpression, increase of activity, and changes in the metabolic network in close proximity of the introduced reaction/pathway, increase product formation, and result here in the majority of fixed carbon channeled into the product L-lactic acid. Notably, under photoautrophic growth conditions no significant amounts of other (carbon-containing) products are excreted by *Synechocystis *[30]*.* The highest yield of lactic acid obtained was 9.29 ± 0.74 mmol/L lactic acid after a two-week period. This was achieved with the strain with the highest gene expression level of the L-*ldh* of *L. lactis* gene plus the overexpression of the PK of *E. faecalis,* SAW041. The same strain shows the highest lactic acid production rate, 0.2512 ± 0.0203 mmol lactic acid/gDW/h, and shows a carbon partitioning into lactic acid of 50.0 ± 5.9%. The highest carbon partitioning ratio into lactic acid observed, 55 ± 2%, was achieved by the strain carrying a single copy of the *B. subtilis ldh*, after engineering of the latter enzyme so that it co-utilizes NADPH. Earlier we reported carbon partitioning rates close to 70% in ethanol production with *Synechocystis *[6]. The main difference between lactic acid and ethanol formation is the K_M_ of the respective pyruvate-utilizing enzymes, which is 0.3 mmol/L for pyruvate decarboxylase employed in ethanol formation, whereas the K_M_ for pyruvate is >1.0 mmol/L for the LDH of *L. lactis *[48] and > 4.0 mmol/L for the LDH of *B. subtilis* both employed in lactic acid formation (see the Results section). We tentatively conclude that these differences in K_M_ values are at the basis of the observed differences in maximal carbon partitioning towards these products.

When considering maximal achievable yields, thermodynamic constraints have to be taken into account. Under “standard conditions,” that is, equimolar amounts of the participating molecules and physiologically relevant conditions (pH = 7.0 and 25°C), the ΔG’° for the reaction pyruvate + NADH = > lactic acid + NAD^+^ is -20.02 kJ/mol [49]. Thus, at chemical equilibrium the ratio of the available co-factors, NAD(P)H/NAD(P)^+^, affects the ratio of pyruvate/lactic acid (compare [6]). This leads to the conclusion that the available amount of intracellular pyruvate (50 μmol/L as reported in [22]) would thus allow one to accumulate up to 175 mmol/L lactic acid in the cells (and hence also in the extracellular medium) at an (assumed) NAD(P)/H co-factor ratio of 1. The thermodynamic driving force of the product-forming reaction further benefits from an (even small) increase in co-factor ratio in favor of the reduced form, as is suggested for the NADPH/NADP^+^ couple in cyanobacteria.

Together, these findings contribute to a better understanding of the design principles to be employed for the construction of cyanobacterial cell factories that can be a modularized at will, depending on the envisioned biosynthetic pathway, for sustainable production of a wide range of chemical commodities.

## Methods

### Bacterial strains and growth conditions

For the cloning procedures, *Escherichia coli* strains XL-1 blue (Stratagene) or EPI400 (Epicentre Biotechnologies) were grown at 37°C in Luria-Bertani (LB) broth or on LB agar.

*Synechocystis* sp. PCC6803 (glucose tolerant, obtained from D. Bhaya, Stanford University, USA) was routinely grown at 30°C in liquid BG-11 medium (Sigma-Aldrich), supplemented with 10 mM TES-KOH (pH 8) and appropriate antibiotics, and incubated with shaking at 120 rpm (Innova 43, New Brunswick Scientific) under moderate intensity white-light illumination (about 35 μE/m^2^/s). The BG-11 agar plates were supplemented with 10 mM TES-KOH (pH = 8), 5 mM glucose, and 0.3% (w/v) sodium thiosulfate.

The growth of *Synechocystis* wild-type and mutant strains was monitored by following OD_730_ (Spectrophotometer Lightwave II, Biochrom) at selected time intervals, and sampling for lactic acid measurements of the supernatant of pelleted cells was performed, essentially as described in [10,14]. Removal of the culture was kept below (and close to) about 1/20 of the total volume at each sampling time point to minimize interfering effects. The conversions to gDW, rate calculations, and the metabolic control analysis have been described earlier in detail [14]. Briefly, in our setup the amount of cells present in 1 liter of OD_730_ of 1.0 corresponds to 0.2 gDW. For the biomass composition we assume an elemental cell composition of C_4_H_7_O_2_N. The sampling time point for enzymatic activity determination and corresponding production rate was guided by the growth behavior of the respective batch cultures (compare Additional file 1: Figure S5).

Natural transformation for genomic integration in *Synechocystis* was performed as described previously [10], using increasing concentrations of antibiotic for growing the transformant to drive segregation. Conjugation of the pDF_LDH plasmid from *E. coli* XL-1 to *Synechocystis* was performed by triparental mating using *E. coli* J53 (pRP4) as the helper strain, essentially as described in [6], using 10 μg/mL streptomycin and 25 μg/mL spectinomycin to select for positive clones. Correct insertion of the genes or plasmid and full segregation were verified by colony PCR with specific primers (Additional file 1: Table S1) and *Taq* DNA polymerase (Thermo Scientific), and subsequent sequencing of the amplified fragment.

Where appropriate, antibiotics were added at the following concentrations: ampicillin (100 μg/mL), kanamycin (20 or 50 μg/mL, for *Synechocystis* and *E. coli*, respectively), streptomycin (10 μg/mL), spectinomycin (25 μg/mL).

### Molecular cloning

Codon optimized sequences encoding the heterologous LDH and/or PK enzymes were synthesized and directly inserted into pHKH001 [10] by GenScript (Piscataway, NJ, USA), flanked by a P*trc1* or P*trc2* promoter, the transcriptional terminator BBa_B0014, and BioBrick-compatible restriction sites. Codon optimization was performed using the OPTIMIZER application and the codon usage table described in [6]. Restriction sites in the coding sequence to be used for further processes were removed using the same OPTIMIZER application [50]. Specific details on plasmids used in this study are listed in Additional file 1: Table S2.

For the construction of SAA026 the plasmid pYW010, a derivative of pHKH020 [14], containing the P*trc1-*driven codon optimized version of *ldh* of *L. lactis* subsp. *cremoris* MG1363 was modified by introducing the gene cassette containing the native *ldh*. Using XbaI/SpeI to remove the *pk* gene cassette from pYW010, and obtaining the *ldh* gene cassette from a precursor to pHKH003, namely pACLDH (a standard BioBrick plasmid backbone which contains the *ldh* gene cassette), the respective cassettes were replaced, creating pDC001.

To construct the reference strain for the PK expressing strains SAW001 (essentially identical to SAA023), we replaced the *pk* gene cassette of pYW001 with a transcriptional terminator (BBa_B0014) by digestion of pYW001 and pSB1AC3_TT (http://partsregistry.org) with XbaI/SpeI and subsequent ligation, which resulted in pAW001.

The conjugative plasmid pDF_LDH was constructed by digestion of pYW001 with PstI/AvrII and pDF_lac [15] (kindly provided by P.R. Jones, Imperial College London, UK) with NsiI/NheI, removing the *lacI* cassette, and subsequent ligation of the fragments, hence inserting the P*trc2-*driven *ldh* of *L. lactis*.

PCR reactions for cloning procedures and amplification prior to sequencing were carried out using the *Pfu* DNA polymerase (Thermo Scientific).

The design of point mutations in the nucleotide sequences of the LDH enzymes was based on the V39R mutation described for the L-LDH [17] of *B. subtilis* strain 168. Since the *B. subtilis* LDH described in that study contains an additional proximal methionine as compared to our *B. subtilis* LDH, we annotated our mutated enzyme with V38R. We assigned the other point mutations after alignment of the amino acid sequences of the different enzymes using Clustal Omega (http://www.ebi.ac.uk/Tools/msa/clustalo/); see also Additional file 1: Figure S4). In the case of the *L. lactis* LDH, the point mutation was applied using overlap extension PCR and specific primers, and using pHKH020 as the template [14]. The sequences encoding the *B. subtilis* LDH and *C. gunnari* LDH were synthesized with their respective point mutation and were mutated back to the wild-type sequence, using the QuickChange protocol with specific primers as described hereafter. First, the genes were amplified from their respective synthesized plasmids introducing NdeI/HindIII for cloning into the overexpression vector pET28b + (Novagen) resulting in an N-terminal His-tag. On the resulting plasmids the site-directed point mutation was introduced by primers in a PCR reaction followed by a DpnI treatment and transformation to *E. coli* XL-1. For overexpression and purification of the wild-type version and for the mutated version V38R of the L-LDH of *B. subtilis* the pET28b + plasmids were further transformed to *E. coli* BL-21 (DE3) (Novagen). To reintroduce the modified genes into the *Synechocystis* integration vector the gene cassettes were amplified from the respective pET28b + construct introducing NdeI/BamHI and replacing the original sequences in pAA028 and pAA30 to create pAA027 and pAA029, respectively.

For the deletion of the gene encoding PEP carboxylase (*ppc; sll0920*), we amplified about 1,000 bp of the regions surrounding *ppc*, using specific anchored primers (Additional file 1: Table S2). The deletion was designed to include the 5’UTR of 55 bp of *ppc*, while leaving the natural asRNA that covers both *ppc* and the downstream gene *sll0921* intact [51]. After digestion, these *ppc*-targeting homologous regions were sequentially cloned into the pHSH vector, thereby replacing the homologous regions that were previously used to target *slr0168.* This resulted in pAW010, now targeting the *ppc* locus. The plasmid pHSH is a derivative of the pHKH001 vector described in [10], thus targeting the neutral site *slr0168,* in which the kanamycin resistance cassette has been replaced by a streptomycin resistance cassette. The streptomycin resistance cassette was amplified from pHP45Ω [52] with primers introducing flanking cloning sites for SalI to replace the kanamycin cassette of pHKH001.

### L-Lactic acid quantification

Supernatant samples of cultures were subjected to the D-/L-lactic acid (Rapid) assay (Megazyme), according to manufacturer protocol, adjusted for use in a 96-well plate as described earlier [10]. Occasionally, the extracellular lactic acid concentration was determined by HPLC analysis. HPLC samples were prepared by treating 500-μL supernatant samples with 50 μL of 35% perchloric acid (Merck), with incubation on ice for 10 minutes and the addition of 27 μL of 7 M KOH (Merck). After vortexing, the precipitate was removed by centrifugation for 5 minutes at 12,000 rpm and subsequent filtering (Sartorius Stedim Biotech, Minisart SRP4, 0.45 μm). Separation of organic acids was achieved by application of a 20-μL aliquot on a Rezex ROA-Organic Acid H^+^ (8%) column (Phenomenex), coupled to a refractive index detector (Jasco, RI-1530), using a flow of 0.5 mL/min and a column temperature of 45°C. The concentration was determined by comparison of the peak with known amounts of lactic acid (Megazyme).

### Preparation of *Synechocystis* soluble lysates and enzymatic activity assays

Cell-free extracts of *Synechocystis* were obtained after harvesting a 10-mL aliquot of a culture at late-exponential growth with an OD_730_ of about 1.0 (Spectrophotometer Lightwave II, Biochrom) by centrifugation (10 min at 4,000 rpm) at 4°C. The resulting cell pellets were disrupted with 100-μm glass beads (Sigma) in pre-chilled buffer, appropriate for the subsequent assays, using a Precellys®24 bead beater (Bertin Technologies). After removal of cell debris by centrifugation (30 min at 14,000 rpm) at 4°C, the protein concentration of these samples was measured using the BCA protein assay (Pierce).

Enzymatic activity assays for LDH were performed as described in [14], measuring consumption of NADH at 340 nm at 30°C. For the L-LDH of *L. lactis*, we used a buffer containing 100 mM Tris-HCl pH 7.2, 2.5 mM MgCl_2_, 3 mM FBP, and 300 μM NADH, starting the reaction with the addition of 30 mM sodium pyruvate. The assay for the the L-LDH of *B. subtilis* was similar; we replaced the buffer with 50 mM phosphate buffer pH 6.5 and no FBP addition.

The PK activity assays were performed in an essentially identical manner, using 50 mM Tris-HCl pH 7.6, 5 mM MgCl_2_, 100 mM KCl, 0.6 mM ADP, 300 μM NADH, about 12U/mL L-LDH (hog muscle, Roche), and optionally 3 mM FBP. PEP (5 mM) was used to initiate the reaction.

### Protein purification

Overnight cultures of *E. coli* BL21 (DE3) harboring pET28b + based plasmids for expression of the wild-type version and for the mutated version V38R of the L-LDH of *B. subtilis*, respectively, were diluted to an OD_600_ of 0.05 in a 200-mL culture. When the cultures reached an OD_600_ of approximately 0.3, expression of the recombinant proteins was induced for 3 hours in the presence of 0.1 mM isopropyl beta-D-thiogalactoside (IPTG). The cells were harvested by centrifugation, and resuspended in 3.5 mL to a concentration of about 50 OD units/mL in a buffer containing 20 mM Na_2_HPO_4_ · 2H_2_O, 500 mM NaCl, and 20 mM imidazol (pH 8.0), and lysed by sonication. Unbroken cells were removed by centrifugation (30 min at 14,000 rpm at 4°C). Subsequently, the soluble lysates were loaded on a washed HisTrap HP Column mounted on an ÄKTA FPLC system (GE Healthcare) at 0.5 mL/min. After extensive washing of the column with filtered washing buffer (20 mM imidazole), the bound protein was eluted with filtered elution buffer, 20 mM Na2HPO4 · 2H2O, 500 mM NaCl, and 500 mM imidazol (pH 8.0), collected in 500-μL fractions. The fractions containing protein were collected and dialyzed overnight against 20 mM Tris-HCl (pH 8.0) at 4°C. The protein purity was verified by CBB-stained SDS-PAGE. The protein concentration was determined using the BCA protein assay (Pierce).

### SDS-PAGE

All obtained samples were dissolved in protein solubilization buffer (50 mM Tris-HCl pH 6.8, 100 mM dithiotreitol, 50 mM EDTA, 2% (w/v) sodium dodecylsulphate, 10% (v/v) glycerol) and boiled at 95°C before continuing with the SDS-PAGE analysis. Protein samples were electrophoresed on SDS-PAGE gels, which were stained with CBB G-250, that is*,* PageBlue Staining solution (Thermo Scientific) according to supplier protocol.

## Abbreviations

CBB: Coomassie Brilliant Blue; FBP: fructose-1,6-bisphosphate; LDH: lactate dehydrogenase; ORF: open reading frame; PEP: phosphoenolpyruvate; PK: Pyruvate kinase; PPC: phosphoenolpyruvate carboxylase; SD: standard deviation; SEM: standard error of the mean; WT: wild type.

## Competing interests

The authors declare that they have no competing interests.

## Authors’ contributions

AA, AW, DC, AV, and VV carried out the experiments and analyzed the data. AA, AW, and KH conceived of the study, participated in its design and coordination, and drafted the manuscript. All authors read and approved the final manuscript.

## Supplementary Material

Additional file 1: Figure S1 Growth and rate of lactic acid production in *Synechocystis* mutants. **Figure S2.** Nonlinear regression employing a rectangular hyperbola (Michaelis-Menten fit) to fit the relation between the relative activity of the heterologously expressed LDH and the resulting rate of lactic acid production. **Figure S3.** K_M_ determination of the purified *B. subtilis* LDHs BsLDH_WT_ and BsLDH_V38R_. **Figure S4.** Alignment of selected LDH enzymes. **Figure S5.** Schematic interpretation of the subsequent growth phases in a batch culture of *Synechocystis*. **Table S1.** Primers used in this study. **Table S2.***E. coli* strains and plasmids used in this study. Click here for file
