# Supplementary material for: Exploring metabolic engineering design principles for the photosynthetic production of lactic acid by Synechocystis sp. PCC6803
Source: Biotechnol Biofuels. 2014 Jun 26;7:99. doi: 10.1186/1754-6834-7-99 (PMC4078008; doi:10.1186/1754-6834-7-99)
Supplement: Additional file 1: Figure S1 — Growth and rate of lactic acid production in Synechocystis mutants. Figure S2. Nonlinear regression employing a rectangular hyperbola (Michaelis-Menten fit) to fit the relation between the relative activity of the heterologously expressed LDH and the resulting rate of lactic acid production. Figure S3. KM determination of the purified B. subtilis LDHs BsLDHWT and BsLDHV38R. Figure S4. Alignment of selected LDH enzymes. Figure S5. Schematic interpretation of the subsequent growth phases in a batch culture of Synechocystis. Table S1. Primers used in this study. Table S2.E. coli strains and plasmids used in this study. [file 1754-6834-7-99-S1.pdf]

Additional file

Exploring metabolic engineering design principles for the photosynthetic production of lactic acid by *Synechocystis* sp. PCC6803

S. Andreas Angermayr†[1], Aniek D. van der Woude†[2], Danilo Correddu[1,2], Angie Vreugdenhil[2], Valeria Verrone[1], Klaas J. Hellingwerf[1,2]*

[†] Equal contribution

[1] Molecular Microbial Physiology Group, Swammerdam Institute for Life Sciences, University of Amsterdam and Netherlands Institute of Systems Biology, The Netherlands

[2] Photanol B.V., Roeterstraat 35, Amsterdam, The Netherlands

[*] Corresponding author at: Swammerdam Institute for Life Sciences, Science Park 904, 1098 XH Amsterdam, The Netherlands, tel: +31-20-5257055, e-mail: K.J.Hellingwerf@uva.nl

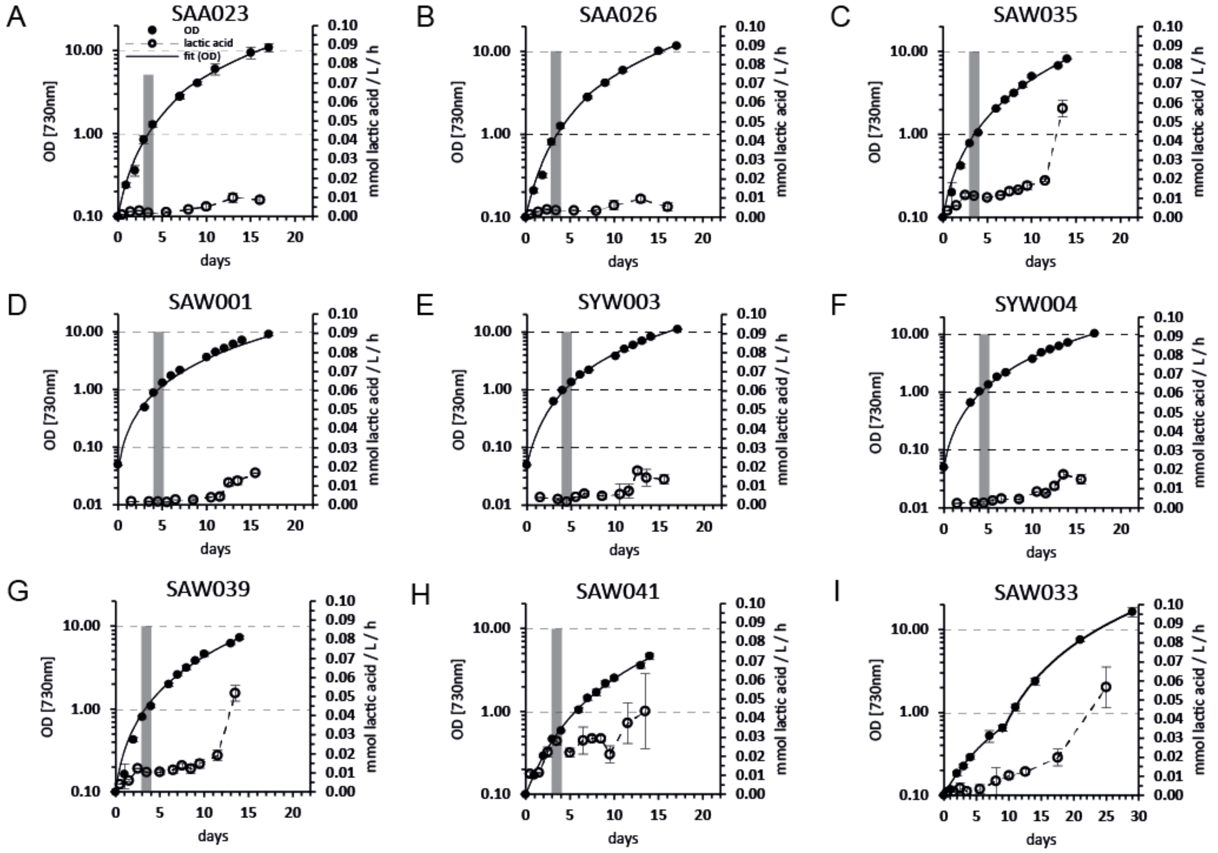

Figure S1. Growth and rate of lactic acid production in *Synechocystis* mutants. (A-I) OD_{730} values were used as a proxy for growth (left ordinate). The rate of lactic acid production is given in mmol lactic acid/L/h (right ordinate). A 2nd order polynomial regression was used to fit the growth data, to achieve smoothing. As introduced in [1] the grey bars indicate the 'linear growth phase' (compare Figure S5) used to correlate enzymatic activity and production rates for the metabolic control analysis (MCA). Error bars represent the SD of biological replicates (n = 3), except for SAW001, SYW003, and SYW004 where n = 2; if error bars are not visible they are smaller than the data point symbol.

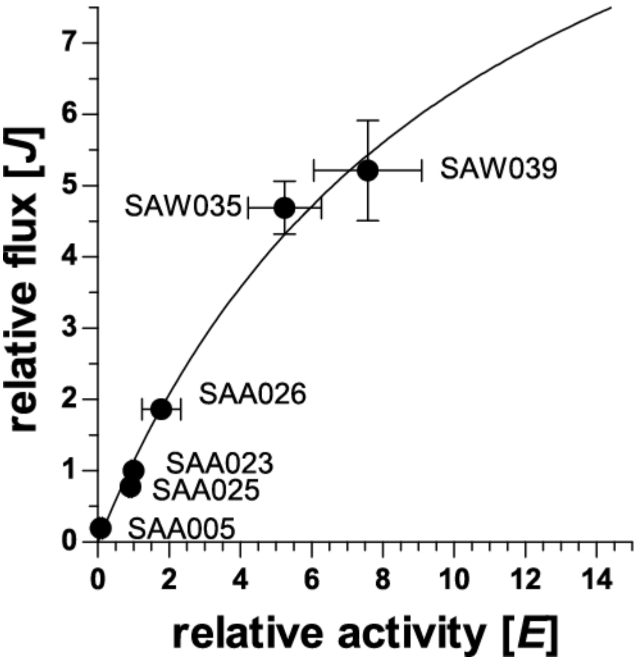

Figure S2. Non-linear regression employing a rectangular hyperbola (Michaelis-Menten fit) [2] **to fit the relation between the relative activity of the heterologously expressed LDH and the resulting rate of lactic acid production.** Prism 5 (GraphPad software) was used with least square optimization. $Y=(B_{max}*X)/(K_d+X)$; $B_{max}=12.99$ (± 3.291); $K_d=10.54$ (±4.109); $R^2=0.9878$.

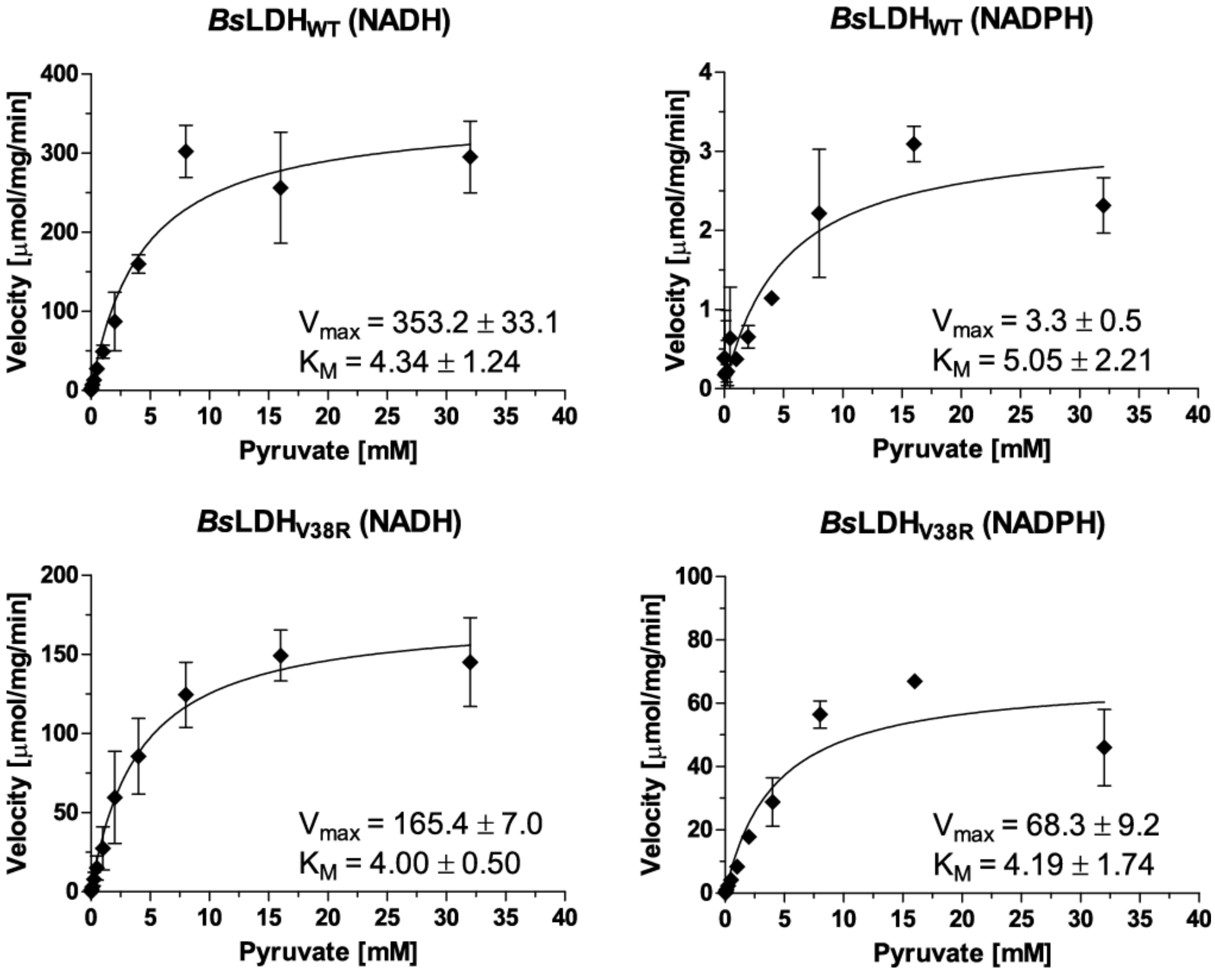

Figure S3. K_M determination of the purified *B. subtilis* LDHs $BsLDH_{WT}$ and $BsLDH_{V38R}$. The V38R mutation leads to an increase of LDH activity with NADPH as the co-factor. The K_M for pyruvate does not change significantly due to this mutation. Errors and error bars represent the SEM (n = 3); if error bars are not visible they are smaller than the data point symbol.

```
Plasmodium_ovale                -----------------------VLVGSGMIGGVMATLIVQKNL-GDVVMFDIVKNMPLG
Champsocephalus_gunnari         MSTKEKLISHVMKEEPVGSRSKVTVVGVGMVGMASAISILLKDLCDELAMVDVMEDKLKG
Lactococcus_lactis              --------------MADKQRKKVILVGDGAVGSSYAFALVNQGIAQELGIVDLFKEKTQG
Bacillus_subtilis168            ---------------MNKHVNKVALIGAGFVGSSYAFALINQGITDELVVIDVNKEKAMG
                                                        ::* * :*   *  :: : :  :: :.*: ::   *

Plasmodium_ovale                KALDTSHTNVMAYSNCQVTGSNTYEDLKGADVVIVTAGFTKAPGKSDKEWNRDDLLPLNN
Champsocephalus_gunnari         EVMDLQHGSLFLK--TKIVGDKDYSVTANSKVVVVTAGARQQEGESRL-----NLVQRNV
Lactococcus_lactis              DAEDLSHALAFTS--PKKIYSADYSDASDADLVVLTSGAPQKPGETRL-----DLVEKNL
Bacillus_subtilis168            DVMDLNHGKAFAP-QPVKTSYGTYEDCKDADIVCICAGANQKPGETRL-----ELVEKNL
                                .. * .*   :            *.    :.:* : :*  :  *::       :*:  *

Plasmodium_ovale                KIMIEIGGHIKNYCPNAFIIVVTNPADVMVQLLHQHSGVSKNKIVGLGGVLDTSRLKYYI
Champsocephalus_gunnari         NIFKFIIPNIVKYSPNCILMVVSNPVDILTYVAWKLSGFPRHRVIGSGTNLDSARFRHLI
Lactococcus_lactis              RITKDVVTKIVASGFKGIFLVAANPVDILTYATWKFSGFPKNRVVGSGTSLDTARFRQAL
Bacillus_subtilis168            KIFKGIVSEVMASGFDGIFLVATNPVDILTYATWKFSGLPKERVIGSGTTLDSARFRFML
                                .*   :  .:     . :::*.:**.*::.    : **. :.:::* *  **::*::  :

Plasmodium_ovale                SQKLNVCPRDVNAHIVGAHGNKMVVLKRYITVGGIPLQEFINNKKI--TDAELDAIFDRT
Champsocephalus_gunnari         GEKLHLHPSSCHAWIVGEHGDSSVPVWSGVNVAGVSLQGLNPQMGTEGDGENWKAIHKMV
Lactococcus_lactis              AEKVDVDARSIHAYIMGEHGDSEFAVWSHANVAGVKLEQWFQENDYL-NEAEIVELFESV
Bacillus_subtilis168            SEYFGAAPQNVHAHIIGEHGDTELPVWSHANVGGVPVSELVEKNDAY-KQEELDQIVDDV
                                .: .     . .* *:* **:. . :    .*.*: :.    :        :   : . .

Plasmodium_ovale                VNTALEIVNYHASPYVAPAAAIIEMAESYLKDLKKVLICSTLLEGQYGHT-GVFGGTPLV
Champsocephalus_gunnari         VDGAYEVIKLKGYTSWAIGMSVADLVESIIKNMHKVHPVSTLVQGMHGVKDEVFLSVPCV
Lactococcus_lactis              RDAAYSIIAKKGATFYGVAVALARITKAILDDEHAVLPVSVFQDGQYGVS-DCYLGQPAV
Bacillus_subtilis168            KNAAYHIIEKKGATYYGVAMSLARITKAILHNENSILTVSTYLDGQYGAD-DVYIGVPAV
                                 : *  ::  :.    . . ::  :.:: :.: . :   *.  :* :*     : . * *

Plasmodium_ovale                LGCNGVEQVFELQLNAEEKKMFDDAI----------------------
Champsocephalus_gunnari         LGNSGLTDVIHMTLKAEEEKQLQKSAETLWGVQKELTL----------
Lactococcus_lactis              VGAEGVVNPIHIPLNDAEMQKMEASGAQLKAIIDEAFAKEEFASAVKN
Bacillus_subtilis168            VNRGGIAGITELNLNEKEKEQFLHSAGVLKNILKPHFAEQKVN-----
                                :   *:    .: *:  * : :  :
```

Figure S4. Alignment of selected LDH enzymes. Protein sequences were aligned using ClustalW2. The highly conserved GXGXXG$_{19}$D motif in the Rossmann fold is indicated with arrows. The valine or (iso)leucine that was changed into an arginine to change cofactor specificity is marked in yellow.

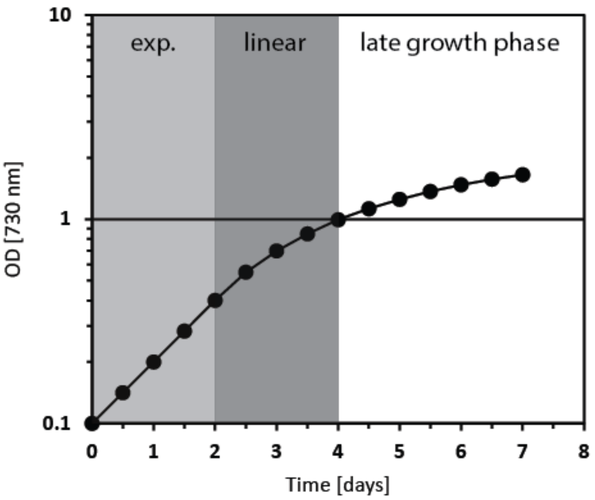

Figure S5. Schematic interpretation of the subsequent growth phases in a batch culture of *Synechocystis*. A typical *Synechocystis* batch culture in BG-11 medium, cultivated at moderate light intensity (for details: see the Material and Methods section) initially shows an exponential growth phase (exp.) which gradually transits into a linear growth phase, because of increased cellular self-shading. After this linear growth phase, cells enter the late growth phase, in which additional limitations (e.g. for carbon or phosphate [3]) take place. We have selected the linear growth phase as the reference phase for calculation of the production characteristics of batch cultures of *Synechocystis*.

Table S1. Primers used in this study.

Name	Sequence*	Additional Information
H1seq_F	AATGGTCCCAAAATTGTC	Colony PCR segregation at *slr0168* insertion
H2seq_R	CTGTGGGTAGTAAACTGGC	Colony PCR segregation at *slr0168* insertion
KanF_back_Seq	TCCCGTTGAATATGGCTC	Colony PCR *slr0168* insertion
coLlLDH_seq_R	AAGTCTTCGGCATCACCTTG	Colony PCR *Llldh* insertion
Synpyk seq R	GGCCTGCATATCTTTAATGG	Colony PCR *Synpk* insertion
Efpyk seq R	GGAATTTCAACACCCATGTC	Colony PCR *Efpk* insertion
ECpyk seq R	GAGGCTTCCAAGATTTCGTC	Colony PCR *Ecpk* insertion
LLpyk seq R	TCGGCGGCTTCAATAATTTC	Colony PCR *Llpk* insertion
SpecR_out_F	CCGAGGCATAGACTGTACCC	Colony PCR *pDF* insertion
Ppc-H1-*SacII*-F	CCCGAGCTCGTTGCCCTCATAGAGCACC	*ppc* deletion
Ppc-H1-*NotI*-R	CCCGCGGCCGCGTACAAGATACTACGGGATT	*ppc* deletion
Ppc-H2-*XhoI*-F	CCCCTCGAGCGTGCGTTACCGTCGTTATTC	*ppc* deletion
Ppc-H2-*KpnI*-R	CCCGGTACCCACGGTTATGCCCGGTTCTG	*ppc* deletion
dPPC_check_F	TTGGCCGGATAAACCAATAA	Colony PCR *ppc* deletion
PPC_check_R	GATTCCGTTGTTGCTCGTTT	Colony PCR *ppc* deletion
ppc_back_R	AGCTCCCCGCAGTAGTTCTT	Colony PCR *ppc* deletion
coLlLDH_mt_L39R_F	AAGAATTGGGTATTGTGGACCGGTTTAAAGAAAAAACCCAAGG	Introduction L39R mutation in *Ll*LDH
coLlLDH_mt_L39R_R	CCTTGGGTTTTTTCTTTAAACCGGTCCACAATACCCAATTCTT	Introduction L39R mutation in *Ll*LDH
coBsLDH_Nde_R	CATATGAACAAGCACGTCAATAAAGT	Cloning of *Bsldh* in pET28b+
coBsLDH_HE_R	GATATCAAGCTTCTGCAGCGG	Cloning of *Bsldh* in pET28b+
coChgLDH_Nde_F	AATCATATGAGCACCAAAGAGAAGTTAAT	Cloning of *Cgldh* in pET28b+
coChgLDH_HE_R	AATGATATCAAGCTTCTGCAGCGG	Cloning of *Cgldh* in pET28b+
coBsLDH_R38V_F	ACGAGTTAGTTGTCATTGATGTAAACAAAGAAAAGGCTATGGG	Removal V38R mutation in *Bs*LDH
coBsLDH_R38V_R	CCCATAGCCTTTTCTTTGTTTACATCAATGACAACTAACTCGT	Removal V38R mutation in *Bs*LDH
coChgLDH_R53V_F	ACGAATTAGCTATGGTGGATGTGATGGAAGACAAGTTGAAGGG	Removal V53R mutation in *Ch*LDH
coChgLDH_R53V_R	CCCTTCAACTTGTCTTCCATCACATCCACCATAGCTAATTCGT	Removal V53R mutation in *Ch*LDH
T7_FW	TAATACGACTCACTATAGGG	Verification of pET28b+
T7_RV	GCTAGTTATTGCTCAGCGG	Verification of pET28b+
coBsLDH_Nde_F	AATCATATGAACAAGCACGTCAATAAAGTG	Cloning of *Bsldh*
coBsLDH_Bam_R	AATGGATCCTTAATTGACTTTCTGTTCAGCGAA	Cloning of *Bsldh*
coChgLDH_Nde_F	AATCATATGAGCACCAAAGAGAAGTTAATCTCC	Cloning of *Cgldh*
coChgLDH_Bam_R	AATGGATCCTTACAAGGTCAATTCTTTTTGCACTC	Cloning of *Cgldh*
Str_Sal_F_R	AATGTCGACGGTGATTGATTGAGCAAGCTTT	Construction of pHSH

* anchoring restriction sites are underlined

Table S2. *E. coli* strains and plasmids used in this study.

**Strain/plasmid**	**Precursor**	*****	**Relevant Insert(s) or resulting genotype****	**Application**	**Reference**
E. coli XL-1 blue		-	*Tet*^{R}	Standard cloning host	Stratagene
E. coli EPI400		-		CopyCutter^{TM} cells for cloning	Epicentre
E. coli BL-21 (DE3)		-		Protein production host	Novagen
pHKH001	pBluescript II SK+	-	*Δslr0168::Kan*^{R}, *Amp*^{R}	Insertion at *slr0168*	[4]
pHKH020	pHKH001	-	Ptrc1::$_{Ll}$*ldh*$_{co}$*::tt::Kan*^{R}	Strain SAA023	[1]
pHKH003	pHKH001	-	Ptrc1::$_{Ll}$*ldh*$_{na}$*::tt::Kan*^{R}	Strain SAA025	[1]
pYW001	pHKH001	S	Ptrc2::$_{Syn}$*pk*$_{co}$*::tt::*Ptrc2::$_{Ll}$*ldh*$_{co}$*::tt::Kan*^{R}	Strain SYW001	This study
pYW002	pHKH001	S	Ptrc2::$_{Bs}$*pk*$_{co}$*::tt::*Ptrc2::$_{Ll}$*ldh*$_{co}$*::tt::Kan*^{R}		This study
pYW003	pHKH001	S	Ptrc2::$_{Ef}$*pk*$_{co}$*::tt::*Ptrc2::$_{Ll}$*ldh*$_{co}$*::tt::Kan*^{R}	Strain SYW003	This study
pYW004	pHKH001	S	Ptrc2::$_{Ec}$*pk*$_{co}$*::tt::*Ptrc2::$_{Ll}$*ldh*$_{co}$*::tt::Kan*^{R}	Strain SYW004	This study
pYW005	pHKH001	S	Ptrc2::$_{Ll}$*pk*$_{co}$*::tt::Ptrc2::*$_{Ll}$*ldh*$_{co}$*::tt::Kan*^{R}	Strain SYW005	This study
pYW010	pHKH001	S	Ptrc1::$_{Syn}$*pk*$_{co}$*::tt::*Ptrc1::$_{Ll}$*ldh*$_{co}$*::tt::Kan*^{R}	pDC001	This study
pAW001	pYW001	C	*tt::Ptrc2::*$_{Ll}$*ldh*$_{co}$*::Kan*^{R}	Strain SAW001	This study
pAV001	pHKH020	C	Ptrc1::$_{Ll}$*ldh*$_{co}$*L39R::tt::Kan*^{R}	Strain SAV001	This study
pSB1AC3		-	*Amp*^{R}, *Cm*^{R}	Standard BioBrick plasmid backbone	http://parts.igem.org
pSB1AC3_TT	pSB1AC3		*BBa_B0014 (tt), Amp*^{R}, *Cm*^{R}	Standard BioBrick plasmid	http://parts.igem.org
pAC_LDH	pSB1AC3	C	Ptrc1::$_{Ll}$*ldh*$_{na}$*::tt*	pAA026	This study
pDC001	pYW010 + pACLDH	C	Ptrc1:: $_{Ll}$*ldh*$_{na}$*::tt::*Ptrc1::$_{Ll}$*ldh*$_{co}$*::tt::Kan*^{R}	Strain SAA026	This study
pAA027	pAA028	C	Ptrc1::$_{Bs}$*ldh*$_{co}$*::tt::Kan*^{R}	Strain SAA027	This study
pAA028	pHKH001	S	Ptrc1::$_{Bs}$*ldh*$_{co}$*V38R::tt::Kan*^{R}	Strain SAA028	This study
pET28b+		-	*Kan*^{R}	Standard cloning and expression vector	Novagen
pETBsLDHw	pETBsLDHm	C	$_{Bs}$*ldh*$_{co}$	His-Tag purification of *Bs*LDH	This study

pETBsLDHm	pAA028	C	${}_{Bs}$*ldh*$_{co}$*V38R*	His-Tag purification of *Bs*LDH$_{V38R}$	This study
pAA029	pAA030	C	P*trc1::*$_{Cg}$*ldh*$_{co}$*::tt::Kan*^{R}	Strain SAA029	This study
pAA030	pHKH001	S	P*trc1::*$_{Cg}$*ldh*$_{co}$*V53R::tt::Kan*^{R}	Strain SAA030	This study
pAA035	pHKH001	S	P*trc1::*$_{Po}$*ldh*$_{co}$*I29R::tt::Kan*^{R}	Strain SAA035	This study
pDF_lac	RSF1010 derivative	-	*lacI*, *Strep*^{R}	Self-replicating plasmid	[5]
pDF_LDH	pDF_lac	C	P*trc2::*$_{Ll}$*ldh*$_{co}$*::tt*, *Strep*^{R}	Strains SAW035, SAW039, SAW041	This study
pHSH	pHKH001	C	*Strep*^{R}	Insertion at *slr0168*	This study
pAW010	pBluescript	C	*Strep*^{R} – homologous regions surrounding *ppc*	*ppc* deletion	This study

* S: directly synthesized and sub-cloned at Genscript (NJ, USA), C: derived from cloning procedures, -: previously described, see reference.

** *Tet*^{R}: tetracyclin resistance, *Kan*^{R}: kanamycin resistance cassette, *Strep*^{R}: streptomycin/spectinomycin resistance cassette, *Amp*^{R}: ampicillin resistance cassette, *Cm*^{R}: chloramphenicol resistance cassette, P*trc1*: trc1 promoter, P*trc2*: trc2 promoter, *tt*: transcriptional terminator (BBa_B0014), *co*: codon-optimized, *na*: native, *Ll: L. lactis*, *Bs*: *B. subtilis*, *Syn*: *Synechocystis*, *Ec: E. coli*, *Ef: E. faecalis*, *Cg: C. gunnari*, *Po: P. ovale.*

References

1. Angermayr SA, Hellingwerf KJ: **On the use of metabolic control analysis in the optimization of cyanobacterial biosolar cell factories**. *J Phys Chem B* 2013, **117**:11169–11175.

2. Fell DA: **Metabolic control analysis: a survey of its theoretical and experimental development.** *Biochem J* 1992, **286**(Pt 2):313–330.

3. Kim HW, Vannela R, Zhou C, Rittmann BE: **Nutrient acquisition and limitation for the photoautotrophic growth of Synechocystis sp. PCC6803 as a renewable biomass source**. *Biotechnol Bioeng* 2011, **108**:277–285.

4. Angermayr SA, Paszota M, Hellingwerf KJ: **Engineering a cyanobacterial cell factory for production of lactic acid**. *Appl Environ Microbiol* 2012, **78**:7098–7106.

5. Guerrero F, Carbonell V, Cossu M, Correddu D, Jones PR: **Ethylene synthesis and regulated expression of recombinant protein in Synechocystis sp. PCC 6803**. *PLoS ONE* 2012, **7**:e50470.
